# Serotonin receptor HTR6-mediated mTORC1 signaling regulates dietary restriction–induced memory enhancement

**DOI:** 10.1371/journal.pbio.2007097

**Published:** 2019-03-18

**Authors:** Ling-Ling Teng, Guan-Ling Lu, Lih-Chu Chiou, Wei-Sheng Lin, Ya-Yun Cheng, Tai-En Hsueh, Yi-Ching Huang, Nai-Hsuan Hwang, Jin-Wei Yeh, Ruey-Ming Liao, Shou-Zen Fan, Jui-Hung Yen, Tsai-Feng Fu, Ting-Fen Tsai, Ming-Shiang Wu, Pei-Yu Wang

**Affiliations:** 1 Graduate Institute of Brain and Mind Sciences, College of Medicine, National Taiwan University, Taipei, Taiwan; 2 Graduate Institute of Pharmacology, College of Medicine, National Taiwan University, Taipei, Taiwan; 3 Neurobiology and Cognitive Science Center, National Taiwan University, Taipei, Taiwan; 4 Graduate Institute of Acupuncture Science, China Medical University, Taichung, Taiwan; 5 Department of Pediatrics, National Taiwan University Hospital Yunlin Branch, Yunlin, Taiwan; 6 Institute of Neuroscience, National Cheng-Chi University, Taipei, Taiwan; 7 Department of Anesthesiology, National Taiwan University Hospital, National Taiwan University, Taipei, Taiwan; 8 Department of Microbiology and Immunology, Indiana University School of Medicine, Fort Wayne, Indiana, United States of America; 9 Department of Applied Chemistry, National Chi Nan University, Nantou, Taiwan; 10 Department of Life Sciences and Institute of Genome Sciences, National Yang-Ming University, Taipei, Taiwan; 11 Department of Internal Medicine, National Taiwan University Hospital, National Taiwan University, Taipei, Taiwan; 12 Center for Systems Biology, National Taiwan University, Taipei, Taiwan; 13 PhD Program in Translational Medicine, National Taiwan University and Academia Sinica, Taipei, Taiwan; 14 Graduate Institute of Neural Regenerative Medicine, College of Medical Science and Technology, Taipei Medical University, Taipei, Taiwan; IMB Mainz, Germany

## Abstract

Dietary restriction (DR; sometimes called calorie restriction) has profound beneficial effects on physiological, psychological, and behavioral outcomes in animals and in humans. We have explored the molecular mechanism of DR-induced memory enhancement and demonstrate that dietary tryptophan—a precursor amino acid for serotonin biosynthesis in the brain—and serotonin receptor 5-hydroxytryptamine receptor 6 (HTR6) are crucial in mediating this process. We show that HTR6 inactivation diminishes DR-induced neurological alterations, including reduced dendritic complexity, increased spine density, and enhanced long-term potentiation (LTP) in hippocampal neurons. Moreover, we find that HTR6-mediated mechanistic target of rapamycin complex 1 (mTORC1) signaling is involved in DR-induced memory improvement. Our results suggest that the HTR6-mediated mTORC1 pathway may function as a nutrient sensor in hippocampal neurons to couple memory performance to dietary intake.

## Author summary

Optimized dietary intake is crucial for maintaining cognitive performance. A mild reduction (between 20% and 40%) in food intake—called dietary restriction (DR) or calorie restriction—has been shown to extend life span and to improve cognitive ability in various species through a mechanism that is not fully understood. Here, we investigate the nutritional basis of DR and find that protein—in particular a single amino acid, tryptophan—is important in limiting the improved memory performance induced by DR. We also observe that DR induces reduced serotoninergic signaling through the suppression of serotonin receptor–mediated mechanistic target of rapamycin complex 1 (mTORC1) signaling in the mouse hippocampus. Whereas genetic and pharmacological inhibition of the 5-hydroxytryptamine receptor 6 (HTR6)–mTORC1 pathway mimics DR-induced structural and electrophysiological changes that are associated with memory enhancement, activation of this pathway diminishes these effects. Our results provide a mechanistic connection linking HTR6 and the mTORC1 pathway that mediates the favorable adaptation of improved memory to reduced dietary intake.

## Introduction

Nutritional status is closely linked to cognitive performance. Whereas high-calorie intake increases the risk for neurodegenerative diseases [1, 2], food shortage can disable costly memory formation in order to favor survival [3]. An adequate but reduced dietary intake, such as dietary restriction (DR; 20%–40% reduction in total daily caloric intake without malnutrition), has been recognized to be the most effective anti-aging intervention, not only improving cognitive performance in elderly humans but also prolonging healthy life span in several model organisms [4, 5]. Studies investigating the nutritional basis of DR benefits have revealed that reduced dietary intake of protein as well as of certain amino acids, including tryptophan, can improve surgical stress resistance and extend life span in rodents [6, 7]. Although the underlying mechanism remains elusive, altered serotonergic signaling is thought to be involved.

Serotonin receptor 5-hydroxytryptamine receptor 6 (HTR6) has been shown to regulate neuronal migration and differentiation during development [8–10] and is also implicated in mental disorders, such as anxiety and depression [11]. HTR6 stimulates Gs and adenylyl cyclase, which are generally known to have a positive influence on cognitive functions [12]. However, accumulating evidence in both rodent and human studies suggests that pharmacological blockade of HTR6 signaling improves memory performance [13–18]. This discrepancy has highlighted alternative pathways that mediate the procognitive effect of HTR6 inhibition. Despite an enhanced corticolimbic release of acetylcholine, glutamate, and monoamines that favors cognitive processes [19], the disrupted recruitment of mechanistic target of rapamycin (mTOR) signaling that occurs upon HTR6 inhibition is postulated as a mechanism mitigating cognitive deficits in animal models of schizophrenia [20].

The mTOR pathway has been shown to integrate signals from nutrients and growth factors, and it further initiates downstream pathways via two distinct protein complexes, mTOR complex 1 (mTORC1) and mTOR complex 2 (mTORC2), which regulate various cellular processes related to growth, differentiation, and metabolic homeostasis [21]. Through its inhibition of the mTOR pathway, rapamycin is one of a few molecules that have been demonstrated to promote memory performance and extend life span in animals [22, 23]. The interplay between dietary manipulation and neuronal control of memory performance has not been thoroughly investigated. We therefore sought to examine the effects of DR on memory performance, and our results demonstrate that dietary tryptophan is a major contributor in limiting DR-induced memory enhancement. The serotonin receptor HTR6 is indispensable for this process, through its modulation of downstream mTORC1 signaling. Our findings thus establish a mechanistic connection between DR and improved memory performance.

## Results

### Chronic DR enhances memory performance in both young and aged mice

**Fig 1:**
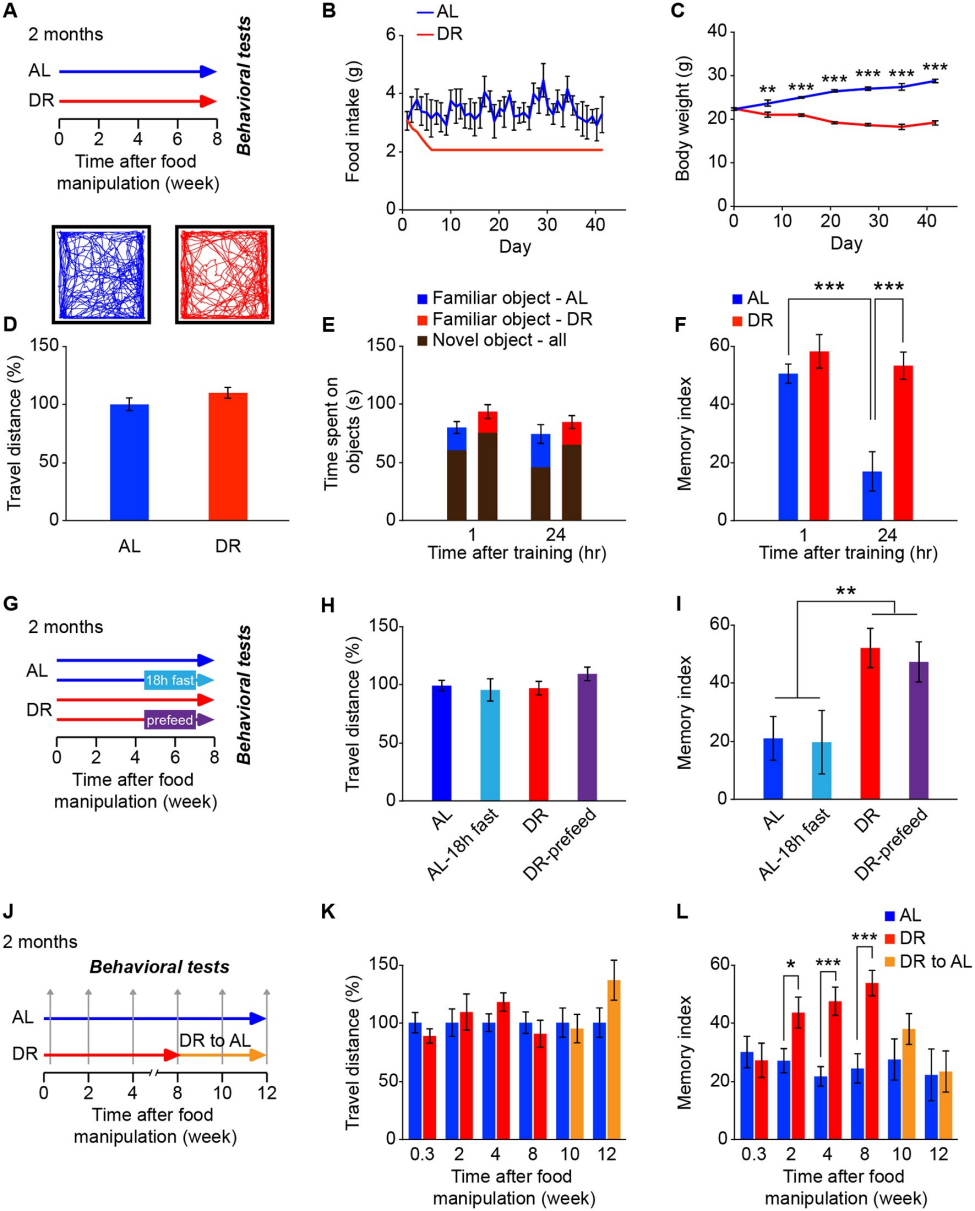
Chronic DR enhances memory performance in young mice. (A–F) The experimental diagram (A), daily food intake (B), body weight (C), representative moving path (upper insets) and travel distance during the open field test (D) and times spent on objects (E), and calculated memory indexes (F) during the NOR test of young mice (2 months old) under dietary manipulations (AL, blue; 60% food intake of AL [DR, red]) for 8 weeks (*n* = 12 mice per group). (G–I) The experimental diagram (G), travel distance during the open field test (H), and calculated memory indexes (I) during the NOR test of young mice under dietary manipulations (18-hour acute fasting and 2-hours-prior prefeeding of AL and DR mice, respectively [*n* = 10 mice per group]). (J–L) The experimental diagram (J), travel distance during the open field test (K), and calculated memory indexes during the NOR test (L) using multiple groups of young mice at different time points throughout the dietary manipulations. Some DR mice were shifted to the AL diet after 8 weeks of DR (*n* = 10–15 mice per group). Data are presented as mean ± SEM. *P < 0.05; **P < 0.01; ***P < 0.001 by Student *t* test, one-way ANOVA, or two-way ANOVA with Fisher’s LSD post hoc test. Underlying data can be found in S1 Data. AL, ad libitum; DR, dietary restriction; LSD, least significant difference; NOR, novel object recognition.

To explore the molecular mechanism underlying DR regulation of memory function, we began by examining the changes in memory performance of young mice (4 months old, Fig 1A–1F) and aged mice (24 months old, S1A–S1F Fig) that had been fed a normal (ad libitum [AL]) or DR diet for 8 weeks. DR mice were fed once a day with a daily food allotment that was 60% of that eaten by the AL animals (Fig 1A and 1B, S1A and S1B Fig). Mice on a DR diet exhibited reduced body weight (Fig 1C and S1C Fig) but did not show noticeable changes in general locomotor activity compared to the AL group in the open field test (Fig 1D and S1D Fig).

Using the novel object recognition (NOR) test, we evaluated the recognition memory of mice at 1 hour or 24 hours after a training session, for both short-term and long-term memory, respectively. The NOR test is based on the innate preference of animals for exploring a novel object over a familiar one, and it is independent of emotional cues [24]. Animals achieving a higher object discrimination index have enhanced memory performance. We found that both young AL and DR mice showed significant memory retention 1 hour after the training session (Fig 1E and 1F). However, young AL mice showed significant memory decline 24 hours after the training session, whereas young DR mice showed sustained memory retention, suggesting that DR mice have improved long-term memory formation (Fig 1E and 1F). Although we also found similar effects of DR on aged mice (S1E and S1F Fig), we focused on young animals for the subsequent mechanistic studies.

We reasoned that appetite might contribute to DR-induced memory enhancement, since DR mice were fed once a day and were fed following training and memory sessions on the days of behavioral tests. To address this issue, we performed 18-hour acute fasting on AL mice and fed the DR mice 2 hours prior to the training session (Fig 1G). Neither 18-hour acute fasting in AL mice nor 2-hours-prior prefeeding in DR mice affected general locomotor activity (Fig 1H) or memory performance (Fig 1I), compared to normal AL and DR mice, respectively. Moreover, we found that the DR-induced memory enhancement required at least 2 weeks of dietary manipulation and that memory performance returned to the normal AL level within 2 weeks following a shift of DR mice to an AL diet (Fig 1J–1L). Thus, chronic DR is essential for enhanced memory performance.

### Dietary protein and tryptophan supplementation limit DR-induced memory enhancement

**Fig 2:**
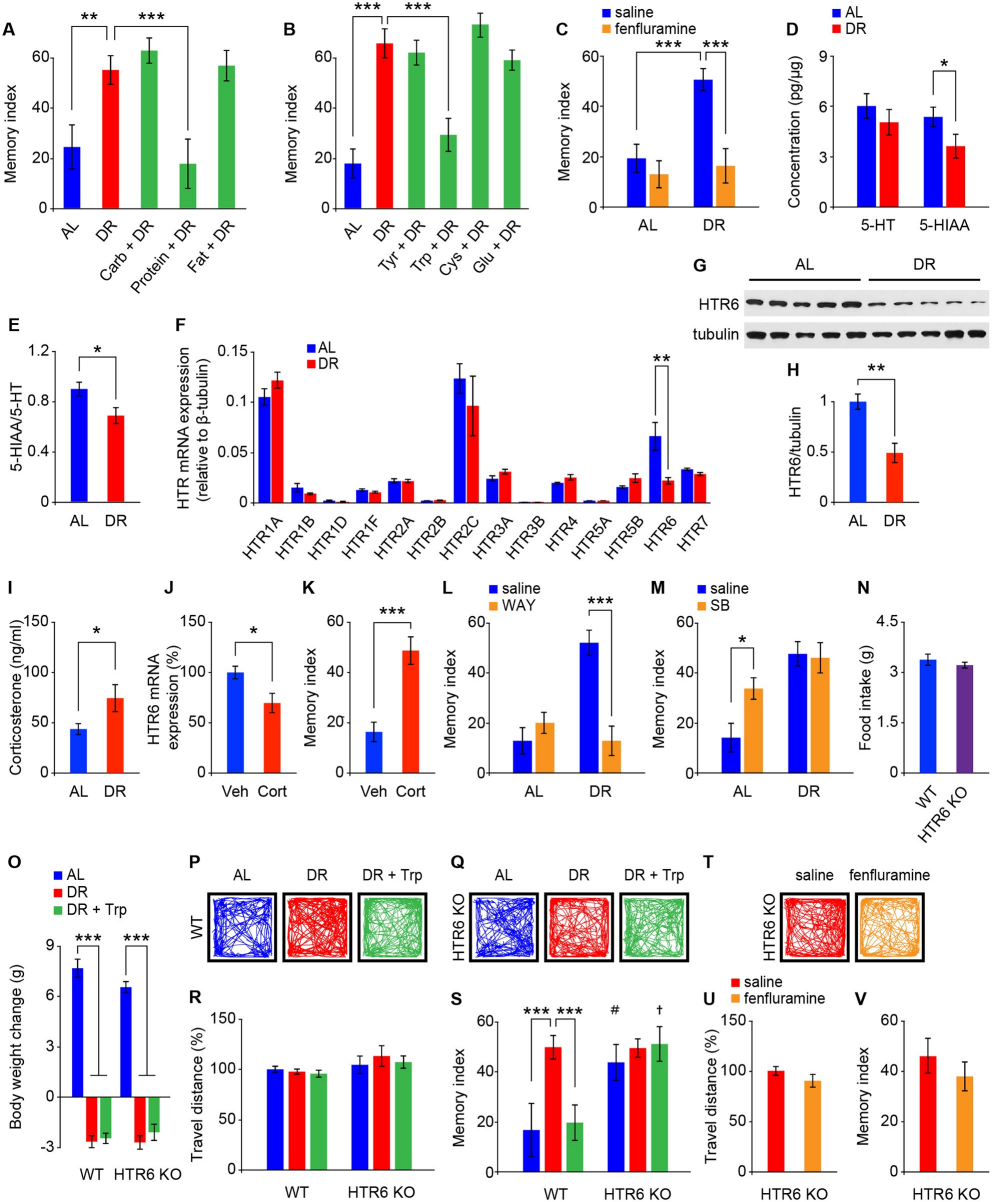
Serotonin receptor HTR6 mediates DR-induced memory enhancement. (A, B) Memory indexes of mice on different diets (AL, DR, and DR plus carb, protein, fat, Tyr, Trp, Cys, or Glu to a level equivalent to AL [*n* = 10–11 mice per group]) for 8 weeks. (C) Memory indexes of AL and DR mice after IP injection of saline or fenfluramine (*n* = 12 mice per group). (D–H) Serotonin and 5-HIAA levels (D), 5-HIAA/5-HT ratios (E), serotonin receptor mRNA levels (F), and representative western blots and quantitative protein expression levels of HTR6 (G, H) in hippocampal tissues of AL and DR mice (*n* = 5–6 per group). (I) An elevated level of serum corticosterone was detected in DR mice compared to the AL group (*n* = 7–9 mice per group). (J–K) HTR6 mRNA expression in mouse hippocampal tissues (*n* = 5 per group) (J) and memory indexes of mice (K) receiving 6 weeks of vehicle (“Veh”) or corticosterone (“Cort”) treatment in the drinking water (*n* = 11 mice per group). (L, M) Memory indexes of AL and DR mice after IP injection of HTR6-specific agonist (WAY) (L) and HTR6-specific antagonist (SB, *n* = 10–12 mice per group) (M). (N) HTR6 KO mice have normal food intake (*n* = 10–11 cages per group). (O-S) The body weight change (O), representative moving path (P, Q), and travel distance during the open field test (R) and calculated memory indexes during the NOR test (S) of WT and HTR6 KO mice after 8 weeks of dietary manipulations (AL, DR, and DR plus Trp to a level equivalent to AL [*n* = 9–14 mice for each group]). (T–V) Representative moving path (T), travel distance during the open field test (U), and calculated memory indexes during the NOR test (V) of HTR6 KO DR mice after IP injection of saline or fenfluramine (*n* = 5–6 mice per group). Data are presented as mean ± SEM. *P < 0.05; **P < 0.01; ***P < 0.001 by Student *t* test, one-way ANOVA, or two-way ANOVA with Fisher’s LSD post hoc test. ^#^*P* < 0.01; †*P* < 0.001 compared to the WT groups by two-way ANOVA with Fisher’s LSD post hoc test. Underlying data can be found in S1 Data. 5-HIAA, 5-hydroxyindoleacetic acid; 5-HT, 5-hydroxytryptamine; AL, ad libitum; carb, carbohydrate; Cys, cysteine; DR, dietary restriction; Glu, glutamate; HTR, 5-hydroxytryptamine receptor; IP, intraperitoneal; KO, knockout; LSD, least significant difference; NOR, novel object recognition; SB, SB 399885 hydrochloride; Trp, tryptophan; Tyr, tyrosine; WAY, WAY 208466 dihydrochloride; WT, wild type.

The major sources of calories in standard mouse food are carbohydrates, proteins, and fats. To identify which of these nutrients from the diet contributes to DR-induced memory enhancement, we compared the recognition memory of mice under DR and under DR supplemented with carbohydrate, protein, or fat to a level equivalent to that of AL feeding (S2 Table). We found that adding back carbohydrate or fat did not affect DR-induced memory retention of mice in the NOR test (Fig 2A), indicating that these constituents do not limit memory performance during DR. In contrast, addition of protein to the DR diet attenuated DR-induced memory performance, bringing it back to a level comparable to that for AL mice (Fig 2A). We next determined which amino acid affected memory function in this context. Glutamate was tested, since it is an amino acid neurotransmitter; tryptophan, tyrosine, and cysteine are precursors for serotonin, catecholamine, and sulfur-containing amino acid biosynthesis in the brain, all of which are known to regulate cognitive function [25, 26]. Our results demonstrated that adding back tryptophan, but not glutamate, cysteine, or tyrosine, attenuated DR-induced memory retention (Fig 2B). Tryptophan alone is thus limiting for memory retention during DR. Although adding back carbohydrate and fat, but not protein or individual amino acids, significantly increased the body weight of DR mice (S2A and S2D Fig), none of these dietary manipulations affected general locomotor activity or total time spent on object exploration, compared to either the AL or DR group in the open field test or NOR test, respectively (S2B, S2C, S2E and S2F Fig). These observations implicated dietary tryptophan in modulation of memory processing, through altered serotonergic signaling in the brain. To test this hypothesis, animals were given an intraperitoneal (IP) injection of fenfluramine, a serotonin-releasing agent [27], approximately 30 minutes prior to the NOR training session. We found that fenfluramine abolished DR-induced memory enhancement (Fig 2C), suggesting that DR may induce reduced serotonergic signaling and that an acute increase in serotonin transmission interferes with DR improvement of memory performance.

### HTR6 mediates DR-induced memory enhancement

To investigate whether DR could indeed affect serotonergic signaling, we measured the levels of serotonin (5-hydroxytryptamine [5-HT]), serotonin metabolite 5-hydroxyindoleacetic acid (5-HIAA), and mRNA expression of serotonin receptors in mouse hippocampal tissues. We found that while DR induced a trend toward reduced serotonin levels, it significantly down-regulated the levels of 5-HIAA in the mouse hippocampus (Fig 2D). The 5-HIAA/5-HT ratio was therefore lower, implying reduced serotonergic activity in the DR mouse hippocampus (Fig 2E). We found that mRNA expression of serotonin receptor HTR6 was significantly lower among all HTRs examined (Fig 2F) and that reduced HTR6 protein expression was also observed in the hippocampus of DR mice (Fig 2G and 2H). Similar results were found in other brain regions involved in memory formation, such as prefrontal cortex (S3A–S3C Fig). DR-induced down-regulation of HTR6 mRNA expression is likely to work through an elevated level of circulating corticosterone, as suggested in previous studies [28, 29]. In agreement with this, we found a higher level of serum corticosterone in DR (compared with AL) mice (Fig 2I), and chronic supplementation with a low dose of corticosterone (10 μg/ml in drinking water for 6 weeks) significantly repressed hippocampal HTR6 mRNA expression (Fig 2J) and improved memory performance (Fig 2K).

We further examined HTR6 involvement in DR-induced memory enhancement in mice by performing IP injection of an HTR6-specific agonist (WAY 208466 dihydrochloride [WAY]) or antagonist (SB 399885 hydrochloride [SB]) approximately 30 minutes before the NOR training session. We found that IP injection of the HTR6 agonist abrogated DR-induced memory enhancement, whereas IP injection of the HTR6 antagonist did not further enhance memory performance of DR mice (Fig 2L and 2M). Moreover, HTR6 antagonist administration alone improved the memory performance of AL mice (Fig 2M), suggesting that HTR6 blockade is beneficial for memory formation. In addition, we obtained HTR6 knockout (KO) mice and confirmed that the HTR6 transcript and protein were undetectable in the hippocampal tissues (S4A and S4B Fig). mRNA expression of the other known HTRs was unchanged in the HTR6 KO mice, indicating absence of any compensatory effect by the other HTRs (S4A Fig). When performing behavioral analyses, we found that HTR6 KO mice showed a higher memory performance under both AL and DR conditions, an improvement comparable to that seen in the wild-type (WT) DR mice, and that the HTR6 KO mice did not exhibit the tryptophan supplementation–or fenfluramine-induced memory attenuation seen in WT DR mice (Fig 2S and 2V). Our data show that the profoundly enhanced memory performance of HTR6 KO mice cannot be attributed to alterations in food intake, body weight, or physical activity (Fig 2N–2R, 2T and 2U). HTR6 KO–induced memory enhancement was also not associated with peripheral glucose metabolism (S4C and S4D Fig) or ketogenesis (S4E Fig), which are known to be involved in DR- or intermittent fasting–induced longevity and neuroplasticity [30, 31].

### HTR6 is required for DR-induced structural alterations and LTP in hippocampal neurons

**Fig 3:**
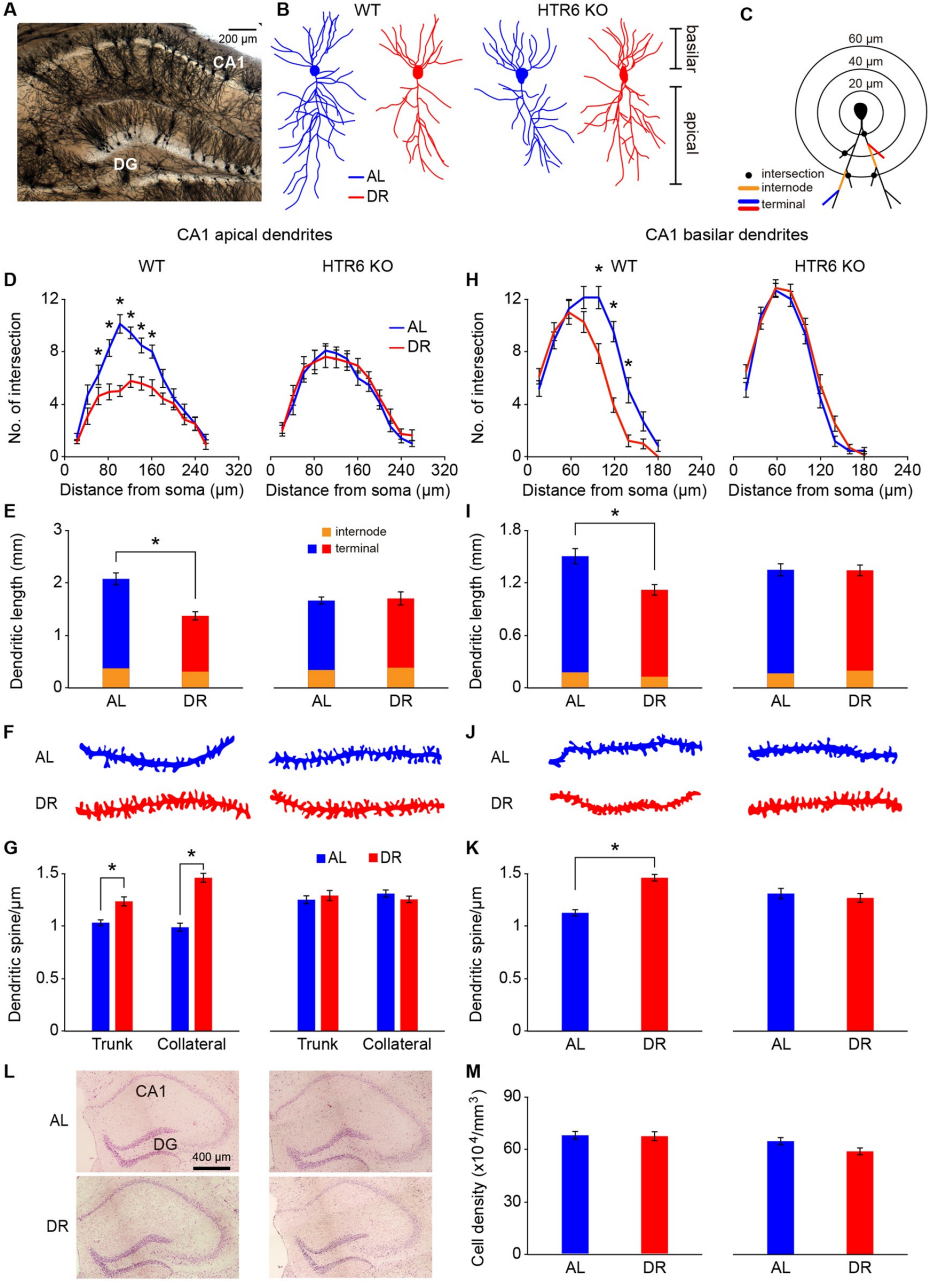
HTR6 is required for DR-induced structural alteration in CA1 hippocampal neurons. Structural analyses of CA1 pyramidal neurons from WT and HTR6 KO mice fed on AL (blue) or DR (red) diet for 8 weeks. (A, B) A representative micrograph (A) and reconstructed CA1 pyramidal neurons (B) following Golgi staining. (C–E) The apical dendritic profile (D) and apical dendritic length (E) of CA1 pyramidal neurons were evaluated using the concentric-ring method of Sholl (C). (F, G) Reconstructed apical collateral dendrites (F) and quantitative spine density (G) of apical trunk and apical collateral dendrites of CA1 pyramidal neurons. (H, I) The basilar dendritic profile (H) and basilar dendritic length (I) of CA1 pyramidal neurons were evaluated using the concentric-ring method of Sholl. (J, K) Reconstructed basilar dendrites (J) and quantitative spine density (K) of basilar dendrites of CA1 pyramidal neurons. (L, M) Representative micrographs (L) and quantitative cell density of CA1 pyramidal neurons (M) following Nissl staining. Data are presented as mean ± SEM (*n* = 15–25 cells, 20 dendritic segments, and 62–82 CA1 regions from 5–8 animals for each group, *P < 0.01 by Student *t* test). Underlying data can be found in S1 Data. AL, ad libitum; DG, dentate gyrus; DR, dietary restriction; HTR6, 5-hydroxytryptamine receptor 6; KO, knockout; WT, wild type.

Two independent gene expression databases, the Allen Brain Atlas of mRNA in situ (www.brainatlas.org) and Gene Expression Nervous System Atlas (GENSAT) of bacterial artificial chromosome–enhanced green fluorescent protein (BAC–eGFP) transgenic mice (www.gensat.org) [32, 33], both indicate that HTR6 is highly expressed in the mouse hippocampus (S5A and S5B Fig). We therefore examined the neuronal morphology of the mouse hippocampus under dietary or genetic manipulations. We used the Golgi-Cox impregnation method and reconstructed the dendritic profile using Neurolucida software. Overall, we found that DR reduced the complexity and dendritic length of the CA1 pyramidal neurons (Fig 3A–3E, 3H and 3I) and dentate gyrus (DG) granule cells (S5C–S5E Fig) of the mouse hippocampus. The spine density of both CA1 pyramidal neurons (Fig 3F, 3G, 3J and 3K) and DG granule cells (S5F and S5G Fig) was significantly increased in the DR mice. However, these DR-induced structural alterations were not observed in the HTR6 KO mice (Fig 3A–3K, and S5C–S5G Fig), suggesting that HTR6 is required for the observed DR-induced structural alterations in hippocampal neurons. Neither DR nor HTR6 KO induced any changes in the neuronal density (Fig 3L and 3M, and S5H and S5I Fig).

**Fig 4:**
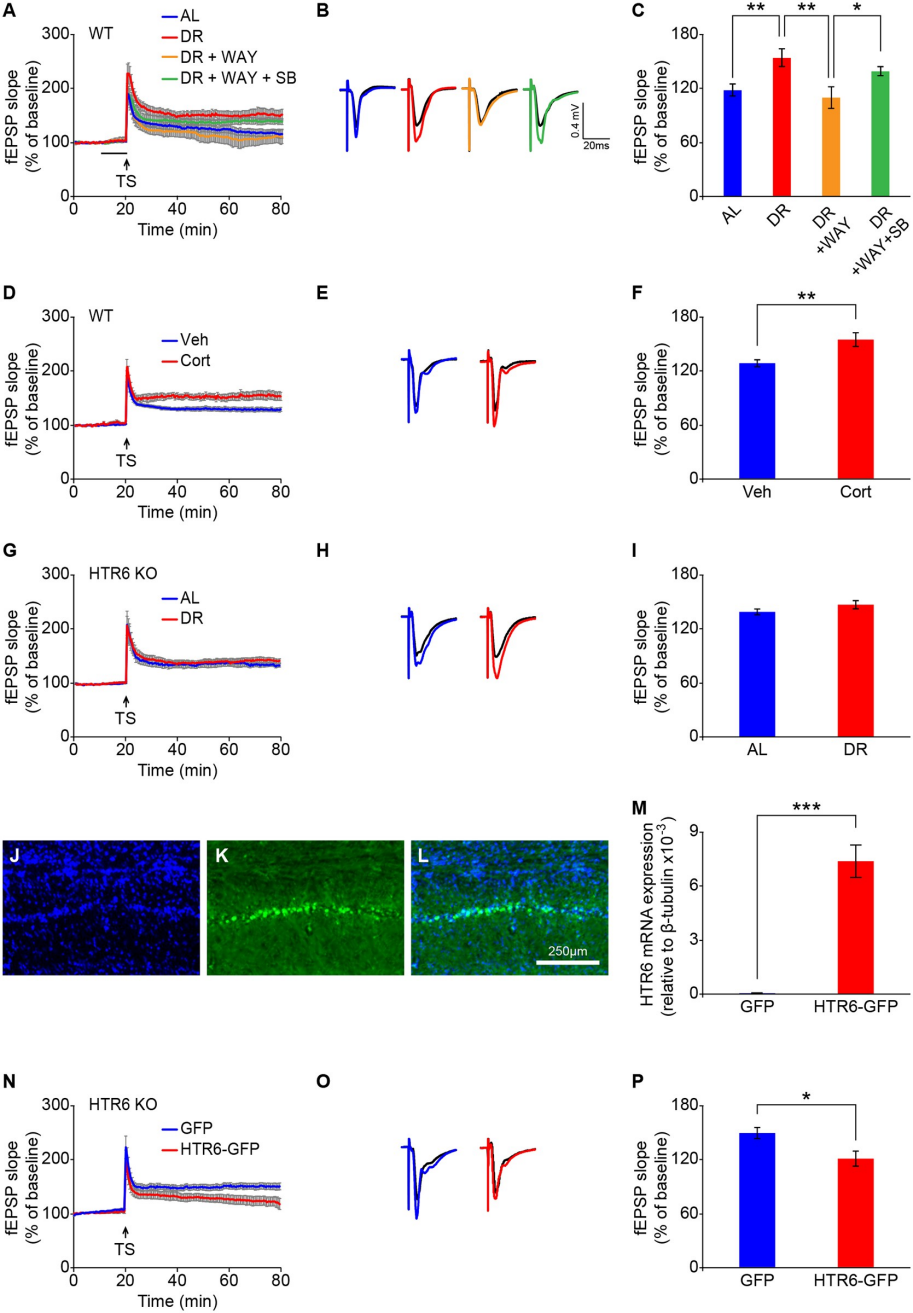
HTR6 is required for DR-induced LTP. LTP of fEPSPs induced by TS was recorded at Schaffer collateral-CA1 synapses of hippocampal slices. Time course of LTP (A, D, G, N), representative traces of average fEPSPs recorded at the baseline (B, E, H, O; black) and during the last 10 minutes of LTP (all other colors), and the average LTP magnitude (C, F, I, P) in various groups. (A–C) Hippocampal slices isolated from WT mice fed on AL or DR diet for 8 weeks were treated with bath application of saline (blue and red), 20 μM WAY (orange), and/or 30 μM SB (WAY + SB, green) 10 minutes prior to TS (horizontal line). (D–F) Hippocampal slices isolated from WT mice receiving 6 weeks of vehicle (“Veh,” blue) or corticosterone (“Cort,” red) treatment in the drinking water. (G–I) Hippocampal slices isolated from HTR6 KO mice fed on AL (blue) or DR (red) diets for 8 weeks. (N–P) Hippocampal slices isolated from HTR6 KO mice transfected with pCMV–GFP (blue) or pCMV–HTR6–GFP (red) plasmid. Data are presented as mean ± SEM (*n* = 6–9 slices in each group). *P < 0.05; **P < 0.01 by Student *t* test or one-way ANOVA with Fisher’s LSD post hoc test. (J–K) Representative micrographs showing that pCMV–HTR6–GFP plasmid was transfected into the hippocampal CA1 region of HTR6 KO mice; GFP signal was detected under a fluorescent microscope (blue: DAPI [J], green: GFP [K], merged image [L]). (M) HTR6 mRNA expression levels of hippocampal tissues from HTR6 KO mice transfected with pCMV–GFP or pCMV–HTR6–GFP plasmid. Data are presented as mean ± SEM (*n* = 6 in each group). ***P < 0.001 by Student *t* test. Underlying data can be found in S1 Data. AL, ad libitum; DG, dentate gyrus; DR, dietary restriction; fEPSP, field excitatory postsynaptic potential; GFP, green fluorescent protein; HTR6, 5-hydroxytryptamine receptor 6; KO, knockout; LSD, least significant difference; SB, SB 399885 hydrochloride; TS, tetanic stimulation; WAY, WAY 208466 dihydrochloride; WT, wild type.

Long-term potentiation (LTP) is a well-recognized synaptic plasticity that reflects higher-order brain functions such as memory. We performed field recordings of Schaffer collateral-CA1 synapses in the hippocampus and found that DR mice had a significantly enhanced LTP compared to AL mice (Fig 4A–4C). This effect was also achieved in AL mice receiving chronic supplementation with corticosterone (Fig 4D–4F). These mice showed improved memory performance similar to that seen in the DR mice (Fig 2K). DR-induced LTP enhancement, however, was attenuated by bath application of the HTR6 agonist during the recordings, and this attenuation was prevented when both the HTR6 agonist and antagonist were present during the recordings (Fig 4A–4C). Consistent with this observation, HTR6 KO mice also exhibited a higher magnitude of LTP, similar to that of DR mice, and DR did not further enhance LTP in HTR6 KO mice (Fig 4G–4I). The DR-induced LTP enhancement seen in both WT and HTR6 KO mice was independent of basal synaptic transmission, as input–output curves obtained in hippocampal slices were similar for AL and DR groups (S5J Fig). We also performed a rescue experiment in the HTR6 KO mice by bilateral injection of HTR6–green fluorescent protein (GFP) or GFP plasmid into the CA1 region of the hippocampus (Fig 4J–4L). We confirmed that HTR6 mRNA was reexpressed in the hippocampus of the HTR6 KO mice (Fig 4M), and the LTP level measured in HTR6–GFP–transfected hippocampal slices from HTR6 KO mice returned to a lower level, similar to that seen in WT AL mice (Fig 4A–4C and 4N–4P). These data together suggest that HTR6 may act downstream of DR to mediate DR-induced synaptic plasticity.

### HTR6-mediated mTORC1 pathway regulates DR-induced memory enhancement and structural alterations

**Fig 5:**
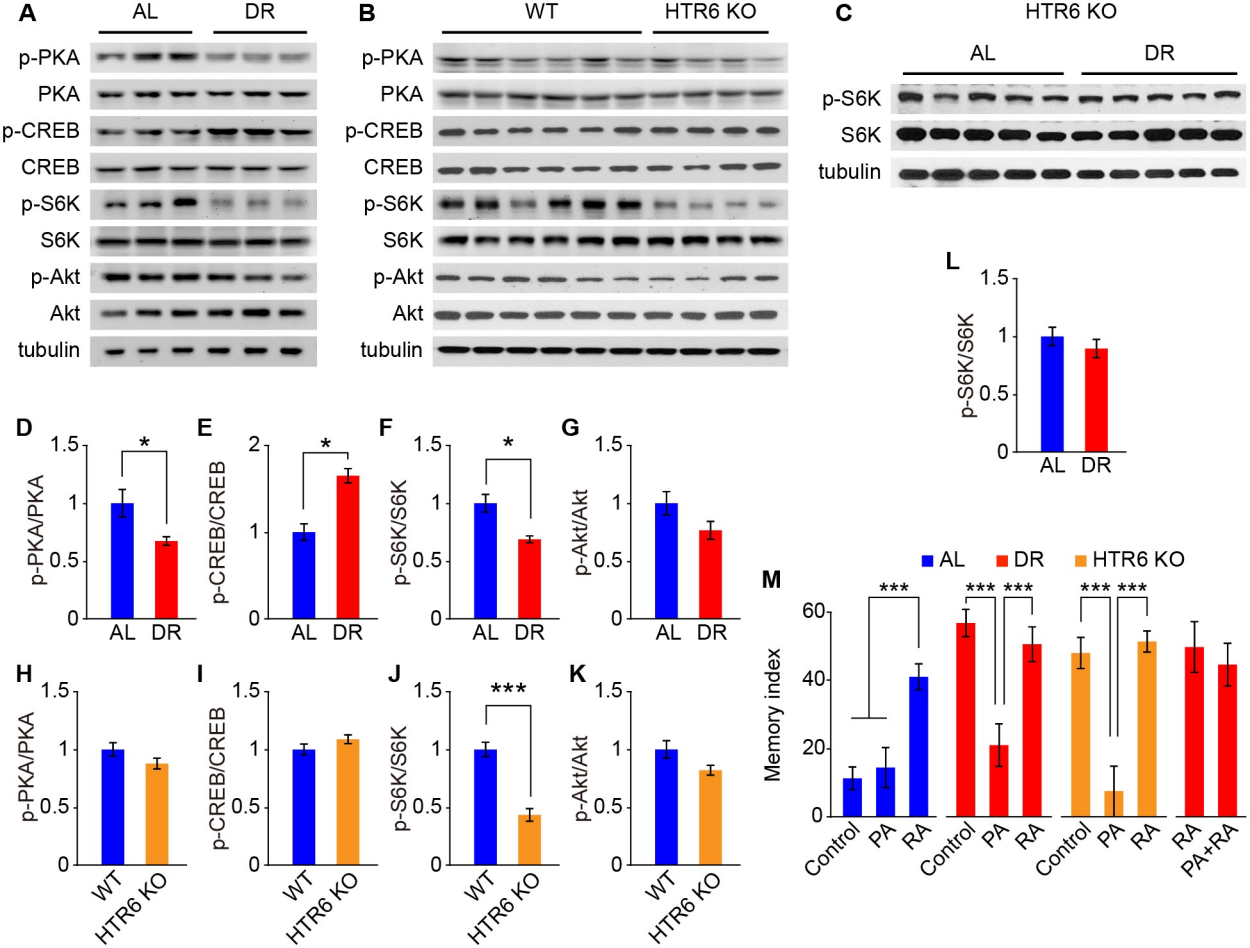
HTR6-mediated mTORC1 signaling regulates DR-induced memory enhancement. (A–L) Representative western blots (A–C) and quantitative results (D–L) of PKA, CREB-1, S6K, and Akt phosphorylation in mouse hippocampal tissues from WT and HTR6 KO mice fed on AL or DR diet (*n* = 3–6 replicates per group). (M) Memory indexes of AL, DR, and HTR6 KO mice on diets supplemented with PA and/or RA for 8 weeks (*n* = 7–12 mice for each group). Data are presented as mean ± SEM. *P < 0.05; ***P < 0.001 by Student *t* test or one-way ANOVA with Fisher’s LSD post hoc test. Underlying data can be found in S1 Data. AL, ad libitum; CREB-1, cAMP-responsive element-binding protein 1; DR, dietary restriction; HTR6, 5-hydroxytryptamine receptor 6; KO, knockout; LSD, least significant difference; mTORC1, mechanistic target of rapamycin complex 1; PA, phosphatidic acid; p-Akt, phosphorylated Akt; p-CREB, phosphorylated CREB; PKA, protein kinase A; p-PKA, phosphorylated PKA; p-S6K, phosphorylated S6K; RA, rapamycin analog; S6K, S6 kinase; WT, wild type.

HTR6 is a Gs-coupled receptor that activates cAMP production upon serotonin stimulation [34]. To identify the downstream effector mediating HTR6 KO–induced memory enhancement, we first examined the cAMP–protein kinase A (PKA)–cAMP-responsive element-binding protein 1 (CREB-1) axis, known to regulate synaptic plasticity and memory [35], in mouse hippocampal tissue. In agreement with previous hypotheses, we found that DR significantly reduced PKA phosphorylation but increased CREB-1 phosphorylation (Fig 5A, 5D and 5E) in mouse hippocampal tissues [36, 37]. However, we observed normal levels of PKA and CREB-1 phosphorylation in the hippocampal tissues of HTR6 KO mice compared to WT mice (Fig 5B, 5H and 5I), suggesting that PKA and CREB-1 activities are uncoupled from HTR6 KO–induced memory enhancement. Since activation of HTR6 has also been shown to recruit and regulate mTOR signaling in transfected human embryonic kidney cells [20], we therefore further explored the activity of mTOR pathways in mouse hippocampal tissues. We found that hippocampal tissue from both DR and HTR6 KO mice showed reduced S6 kinase (S6K; downstream of mTORC1) phosphorylation, but not Akt (downstream of mTORC2) phosphorylation, compared to tissue from AL or WT mice (Fig 5A, 5B, 5F, 5G, 5J and 5K). Additionally, DR did not further reduce S6K phosphorylation in the HTR6 KO mice (Fig 5C and 5L), indicating that DR and HTR6 affect mTORC1 activity through a common pathway. To demonstrate whether mTORC1 signaling may mediate DR- and HTR6 KO–induced memory enhancement in mice, we performed behavioral tests and found that supplementation of food with an mTORC1 activator (phosphatidic acid [PA]) [38] attenuated DR- and HTR6 KO–induced memory performance (Fig 5M). On the other hand, supplementation of food with an mTORC1 inhibitor (everolimus, rapamycin analog [RA]) mimicked but did not further enhance the memory performance of DR and HTR6 KO mice (Fig 5M). Supplementation with the mTORC1 inhibitor also prevented mTORC1 activator–induced memory impairment in the DR mice (Fig 5M). Neither mTORC1 activator nor inhibitor treatment affected feeding behavior, body weight, or locomotor activity of mice (S6A–S6C Fig).

**Fig 6:**
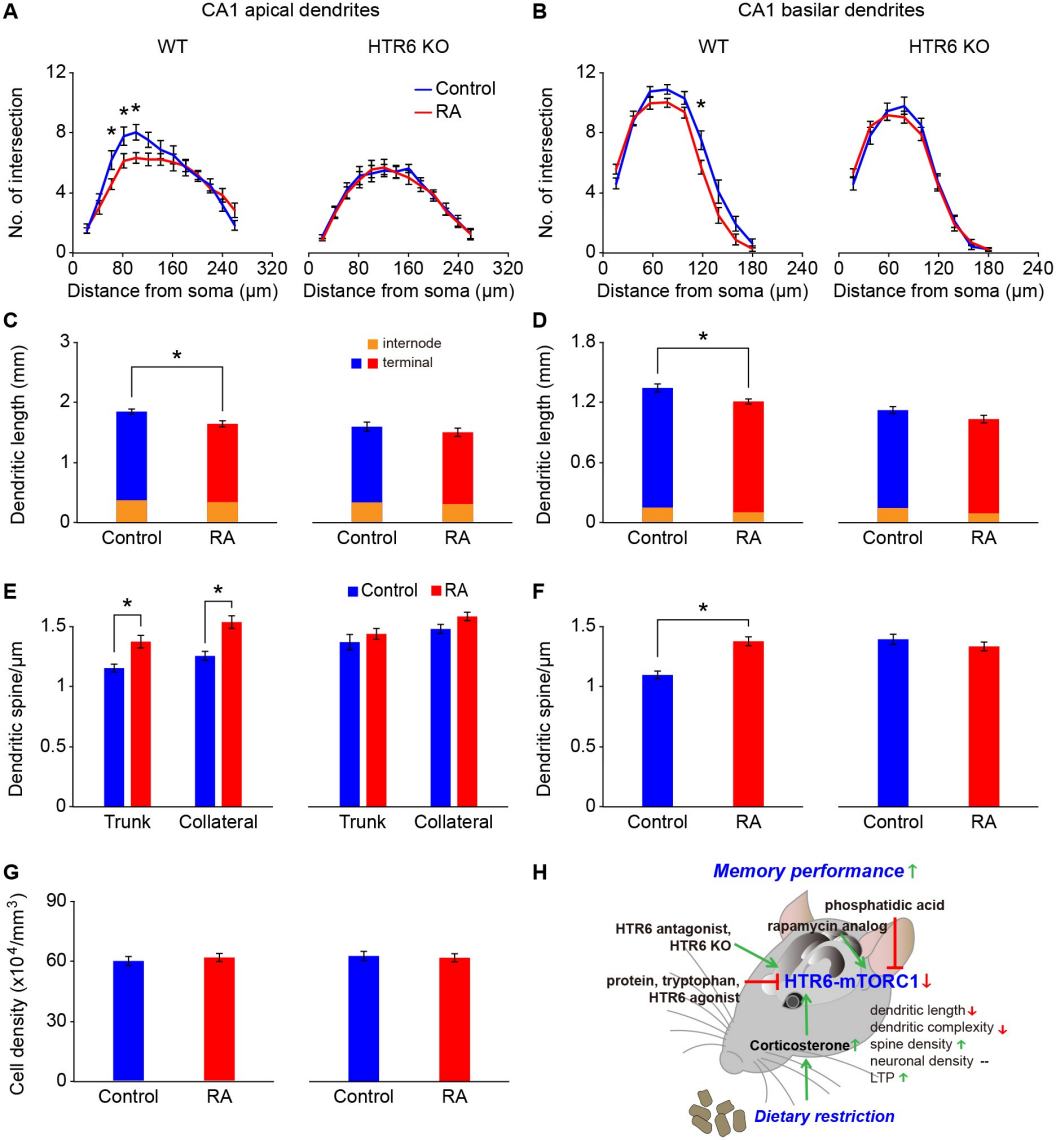
HTR6-mediated mTORC1 pathway mediates DR-induced structural alterations. Structural analyses of CA1 pyramidal neurons from WT and HTR6 KO mice fed on an AL diet supplemented with or without RA for 6 weeks. (A, C) The apical dendritic profile (A) and apical dendritic length (C) of CA1 pyramidal neurons were evaluated using the concentric-ring method of Sholl. (E) Quantitative spine density of apical trunk and apical collateral dendrites of CA1 pyramidal neurons. (B, D) The basilar dendritic profile (B) and basilar dendritic length (D) of CA1 pyramidal neurons were evaluated using the concentric-ring method of Sholl. (F) Quantitative spine density of basilar dendrites of CA1 pyramidal neurons. (G) Quantitative cell density of CA1 pyramidal neurons following Nissl staining. Data are presented as mean ± SEM (*n* = 20 cells, 23–25 dendritic segments, and 65 CA1 regions from 5–6 animals for each group, *P < 0.05 by Student *t* test). (H) A working model for DR-induced memory improvement. In this model, DR results in an elevated level of circulating corticosterone that represses HTR6 expression and down-regulates mTORC1 signaling in hippocampal neurons. This further leads to enhanced memory performance. However, this pathway can be reversed by an HTR6 agonist and dietary protein (tryptophan) or PA supplementation. HTR6 KO, HTR6 antagonist, and RA administration all can mimic DR-induced memory improvement. Although DR reduces the complexity and dendritic length of hippocampal neurons, the presence of increased spine density and LTP formation supports our findings of improved memory performance in mice. Underlying data can be found in S1 Data. AL, ad libitum; DR, dietary restriction; HTR6, 5-hydroxytryptamine receptor 6; KO, knockout; LTP, long-term potentiation; mTORC1, mechanistic target of rapamycin complex 1; RA, rapamycin analog; WT, wild type.

Similar to the results in DR mice, WT AL mice fed a diet supplemented with an mTORC1 inhibitor also showed reduced dendritic complexity, reduced dendritic length, increased spine density, and normal neuronal density of the CA1 pyramidal neurons (Fig 6A–6G). However, these mTORC1 inhibitor–induced structural alterations were not observed in the HTR6 KO mice (Fig 6A–6F), suggesting that reduced HTR6-mediated mTORC1 signaling is essential for imitating the DR-induced structural alterations in hippocampal neurons.

## Discussion

In this study, we examined the nutritional basis and mechanistic regulation of DR in enhancing normal brain function. We found that a chronic and constant regimen of DR can effectively improve memory performance of mice through negative modulation of HTR6- and mTORC1-mediated serotonergic signaling. Our data show that tryptophan supplementation limits DR-induced memory enhancement, and it is therefore reasonable to assume that reduced serotonergic signaling in the brain of DR mice may be responsible for this effect. Although we observed only a trend toward low levels of 5-HT or 5-HIAA in DR brain tissues, a significantly reduced 5-HIAA/5-HT ratio could indicate a dampened serotonin turnover rate, a reflection of reduced serotonergic activity in the brain [39]. These results are consistent with previous findings, which showed that DR suppresses serotonergic activity in the brain [40, 41]. Our data also showed that fenfluramine administration abolished the beneficial effect of DR on memory performance of mice, further implicating reduced serotonergic signaling in the DR brain tissues. This hypothesis is strongly supported by our findings of reduced HTR6-mediated mTORC1 signaling in the hippocampal tissue of DR mice. Extracellular and intracellular levels of serotonin will need to be measured in future studies in order to confirm the role of DR in serotonin metabolism in the brain.

Current opinion regards DR as a form of intermittent metabolic switching (IMS) in which the brain experiences transitional cycles of utilizing carbohydrates and ketones as major energy sources. Although the mechanisms for IMS-induced neuroplasticity remain to be established, both peripheral circulating signals and intrinsic neuronal network pathways are proposed [31]. Our results suggest that the elevated level of circulating corticosterone, but not altered glucose metabolism or ketogenesis, mediates DR-induced memory enhancement through repressed hippocampal HTR6 expression and consequently reduced mTORC1 signaling. The brain is considered to be an important target for corticosterone, since two types of receptors, the type I high-affinity mineralocorticoid receptor (MR) and the type II lower-affinity glucocorticoid receptor (GR), are highly expressed in the hippocampus and many other brain regions that are involved in multiple cognitive processes [42, 43]. Previous studies have demonstrated a biphasic effect of corticosterone on cognitive function. Whereas enhanced memory and LTP occur when the level of corticosterone is mildly increased (predominantly MR activation), impaired memory and LTP appear when the corticosterone level is greatly increased (both MR and GR activation) [44–46]. This biphasic effect of corticosterone is also observed in the regulation of hippocampal neurogenesis, which is known to be associated with DR-induced memory formation [47, 48]. A low dose of corticosterone treatment, similar to that used in our study, does not induce stress responses in mice [49], but the improved memory performance and LTP observed in our study are largely in agreement with previous studies discussed above.

HTR6 was originally identified as a Gs-coupled receptor that activates cAMP production upon serotonin stimulation [34], and recent characterization of intracellular binding partners for HTR6 have also revealed other ligand-dependent and ligand-independent pathways that regulate a number of cellular functions [10, 20, 50]. Our data are in line with the notion that mTORC1 functions as an alternate downstream effector of HTR6 [20] and that this pathway may further regulate structural alteration and neuronal plasticity in aiding memory performance. A possible linkage between DR, HTR6, and the cAMP–PKA–CREB-1 axis in memory regulation is suggested by a previous study demonstrating a role for CREB-1 in mediating DR-induced neuronal plasticity, memory, and social behavior [36]. However, the concept of an up-regulated HTR6–cAMP–PKA–CREB-1 axis in memory enhancement contradicts the generally accepted idea that HTR6 inactivation is promnemonic [13–20]. Our findings that chronic DR reduced PKA phosphorylation but increased CREB-1 phosphorylation in mouse hippocampal tissue indicate that PKA and CREB-1 have more dynamic interactions during memory formation that require further investigation. The key feature of the cAMP–PKA–CREB-1 pathway is its transient activation in response to stimulation of Gs-coupled receptors, which regulate transcription, with rates peaking between 30 minutes and 1 hour [51, 52]. In this study, we measured the steady state of PKA and CREB-1 phosphorylation following chronic dietary manipulation in both WT and HTR6 KO mice. These data do not reflect acute cellular responses of PKA and CREB-1 phosphorylation during memory behaviors. A lower intrinsic PKA phosphorylation level in the brain tissues of DR mice could imply a higher efficacy in activating downstream signaling molecules upon transient stimulation, which may be critical during memory formation in living animals. In addition to the PKA pathway, CREB-1 can also be activated through other kinases to mediate neuronal activity, growth factor signaling, and stress responses [53–58]. It is of particular interest that DR up-regulates N-methyl D-aspartate receptor and brain-derived neurotrophic factor/tropomyosin receptor kinase B signaling, which may result in the increased CREB-1 activity observed in the brain of the DR mice [30, 59]. Accordingly, creating an in vitro cell culture environment mimicking chronic DR and a real-time monitor for protein (kinase) activity during memory behaviors will be important for future studies to establish which signaling networks are influenced by DR.

mTOR exerts a critical role in the regulation of dendritic protein synthesis, which is essential for long-lasting synaptic plasticity [60]. Current concepts of neuroplasticity and memory regulation refer to mTOR as a rheostat rather than an on–off switch. Whereas acute and complete inhibition of mTOR abolishes synaptic plasticity, chronic partial reduction of mTOR signaling may result in DR-mimicking effects, leading to the enhanced memory performance in animals observed in this study and consistent with previous reports [61, 62]. At a structural level, mTOR is involved in the regulation of dendritic formation and axon elongation, as well as synaptic pruning, all of which are critical for normal brain development [63, 64]. Our analyses of Golgi staining revealed HTR6-dependent structural alterations in DR hippocampal neurons, including reduced dendritic complexity and dendritic length but increased spine density. These results suggest that nutritional restriction, such as DR, with reduced HTR6-mediated mTOR signaling may minimize the dendritic size and complexity of neurons but that increased spine density may compensate for these changes by permitting more efficient communication among neurons. It is also worth noting that HTR6 has been shown to regulate neuronal differentiation through constitutive interaction with cyclin-dependent kinase 5 (Cdk5), which is known to control cytoskeletal dynamics involved in dendritic spine morphogenesis and neurite growth, as well as neuronal migration [65]. Although examination of the role of HTR6 in neuronal morphogenesis is not our primary focus, the current findings provide added evidence for a connection between diet and dendritic organization in neurons. Future examination of the interactions among HTR6, mTOR, and Cdk5 would certainly broaden our understanding of nutritional control in dendritic arborization and spine formation.

In summary, we propose a mechanism that explains DR-induced memory enhancement and identify serotonin receptor HTR6, in association with the mTORC1 pathway, as playing a pivotal role in this process (Fig 6H). Our dietary, pharmacological, and genetic manipulations point to attenuated HTR6-mediated mTORC1 signaling in the brain of DR mice, and our results show that interventions that interfere with this pathway compromise the favorable adaptation of memory functions to reduced dietary intake. These results are also supported by previous findings of increased hippocampal spine density and LTP formation in DR animals [36, 66], even in the presence of reduced dendritic complexity and dendritic length, as observed in this study.

## Materials and methods

### Ethics statement

All experimental protocols followed local animal ethics regulations and were approved by the National Taiwan University College of Medicine and College of Public Health Institutional Animal Care and Use Committee (approval no. 20120262).

### Animal and food manipulations

C57BL/6 mice were obtained from the Laboratory Animal Center, National Taiwan University College of Medicine. HTR6 KO mice (B6;129S5-*Htr6*^*tm1Lex*^/Mmucd) were obtained from the Mutant Mouse Resource & Research Centers at University of California, Davis and were backcrossed to the C57BL/6 mouse background for 10 generations. Each mouse was genotyped using gene-specific primers (S1 Table) for polymerase chain reaction (PCR) and gel electrophoresis as described in our previous study [67]. Male mice were used in this study, and all mice were group-housed (2–5 mice per cage) and maintained in an animal room at a controlled temperature of 22–24 °C and 50%–55% humidity, under a 12-hour light/dark cycle. Mice were fed once per day, at the beginning of the dark phase, with purified rodent diet AIN-93G powder (MP Biomedicals) supplemented with different nutrients as indicated in each experiment (S2 Table). PA (30 g/kg; Avanti) and RA (15 mg/kg; everolimus, Tocris Bioscience) were added to AIN-93G as a daily diet for some experiments. Corticosterone (10 μg/ml, Sigma) was added to the drinking water of mice. Food intake and change in body weight of mice were monitored regularly. All of the following behavioral, morphological, and electrophysiological analyses were done blind with respect to the diet and genotype of the mice.

### Behavioral tests

All behavioral tests were performed in the dark phase. The open field test and NOR test were performed as described previously [68, 69]. We calculated the object discrimination index, in order to measure the memory performance of mice, by subtracting the time spent on exploring the familiar object from the time spent on exploring the novel object and dividing by total time spent exploring both objects. Fenfluramine (5 mg/kg, Sigma), WAY (10 mg/kg; Tocris Bioscience), and SB (10 mg/kg; Tocris Bioscience) were IP injected into mice 30 minutes before the NOR training session.

### mRNA quantification

Total RNA was prepared from hippocampal tissue of each mouse using the NucleoSpin RNA Kit (Macherey-Nagel), and cDNA was prepared using oligo-d(T)_15_ (Invitrogen) and SuperScript III reverse transcriptase (Invitrogen), as described previously [70]. Quantitative PCR was carried out using a StepOnePlus Real-Time PCR System (Applied Biosystems), SYBR Green Master Mix (Fermentas), and gene-specific primers (S1 Table).

### Glucose tolerance test, insulin tolerance test, and beta-hydroxybutyrate measurements

Four-month-old mice were fasted for 6 hours, and blood glucose concentration was measured at 0, 30, 60, 90, 120, and 180 minutes following an IP injection of glucose (2 g/kg, Sigma) or insulin (0.75 unit/kg, Sigma). Blood samples from nonfasted and 6-hour-fasted mice were collected for beta-hydroxybutyrate measurements. The concentrations of blood glucose and beta-hydroxybutyrate were measured using FreeStyle Optium Neo Blood Glucose and Ketone Monitoring System (Abbott Diabetes Care).

### Serotonin and corticosterone measurements

Serotonin (Abcam) and 5-HIAA (BioVision) concentration in the mouse brain tissues were measured using enzyme-linked immunosorbent assays, following the manufacturer’s instructions. Total protein level was quantified using the Bradford protein assay (Bio-Rad). Corticosterone concentration in the mouse serum samples was determined using a corticosterone enzyme-linked immunosorbent assay, following the manufacturer’s instructions (Enzo Life Sciences). Blood samples were collected during the dark phase (around 6 to 8 PM).

### Golgi staining, Nissl staining, and dendritic analyses

Morphologic features of CA1 pyramidal neurons and granule cells in the DG were visualized using the FD Rapid GolgiStain kit following the manufacturer’s protocol (FD NeuroTechnologies). Dendritic morphology and spine density were reconstructed and analyzed using Neurolucida software (MBF, Bioscience). For neuronal density analysis, 7-μm coronal sections of mouse brain were stained with 0.1% cresyl violet, and every 20th section from dorsal to ventral hippocampus was examined using a 63x oil-immersion objective lens on a photomicroscope (Zeiss Axio Imager 2). The neuronal density of the CA1 pyramidal neurons and DG granule cells was quantified using Image J software.

### Electrophysiology

Extracellular recordings of field excitatory postsynaptic potentials (fEPSPs) at Schaffer collateral-CA1 synapses in mouse hippocampal slices were performed with a MED64 multichannel recording system equipped with a data acquisition and analysis program (Alpha MED sciences), as described in our previous study [71]. LTP was induced by tetanic stimulation (TS) at 100 Hz for 1 second. In each slice, fEPSPs were monitored for at least 30 minutes to obtain stable fEPSPs. The slopes of fEPSPs recorded for the following 10 minutes were averaged and taken as the baseline. LTP magnitudes were obtained by the average slope of the least 20 fEPSPs (10 minutes) recorded following TS and expressed as the percentage of baseline fEPSP slope. Brain slices were treated with saline, 20 μM WAY, and/or 30 μM SB 10 minutes prior to TS. For rescue experiments in the HTR6 KO mice, pCMV–GFP or pCMV–HTR6–GFP plasmid (0.5 μg) was mixed with BrainFectIN transfection regent and bilaterally injected into the CA1 regions of the mouse hippocampus (ML: ±1.5 mm, AP: −2 mm, DV: −1.5 mm). Murine HTR6 was cloned into pCMV–GFP, a gift from Connie Cepko (Addgene plasmid #11153). LTP measurements were performed 5–7 days after transfection.

### Western blot analysis

Brain tissues were lysed in radioimmunoprecipitation assay buffer (Thermo Fisher Scientific). Proteins were then separated by SDS-PAGE and transferred to PVDF membranes (Millipore) using standard procedures [72]. The antibodies used were rabbit anti-HTR6 (1:500, Abcam #ab103016), rabbit anti-phospho-S6K (Thr389, 1:1,000, Cell Signaling Technology #9205), rabbit anti-S6K (1:1,000, Cell Signaling Technology #2708), rabbit anti-phospho-Akt (Ser473, 1:1,000, Cell Signaling Technology #9271), rabbit anti-Akt (1:1,000, Cell Signaling Technology #9272), rabbit anti-phospho-PKA (Thr197, 1:1,000, Cell Signaling Technology #4781), rabbit anti-PKA (1:1,000, Cell Signaling Technology #4782), rabbit anti-phospho-CREB (Ser133, 1:1,000, Millipore #06–519), rabbit anti-CREB (1:1,000, Millipore #AB3006), and mouse anti-α tubulin (1:10,000, GeneTex #GTX628802). Protein signals were visualized with horseradish peroxidase–conjugated secondary antibodies and ECL reagent (Thermo Fisher Scientific). Quantification of immunoblots was conducted with Image J software.

### Statistical analysis

All data are expressed as mean ± SEM and were examined by Student *t* test, one-way ANOVA, or two-way ANOVA with Fisher’s LSD post hoc test (StatPlus:mac). The statistical details of experiments can be found in the figure legends.

## Supporting information

S1 Fig DR enhances memory performance in aged mice. (A) The experimental diagram, (B) daily food intake, (C) body weight, (D) representative moving path (upper insets) and travel distance during the open field test, and (E) times spent on objects and (F) calculated memory indexes during the NOR test of aged mice (22 months old) under dietary manipulations (AL, blue; 60% food intake of AL [DR, red]) for 8 weeks (*n* = 9 mice per group). Data are presented as mean ± SEM. *P < 0.05; **P < 0.01 by Student *t* test. Underlying data can be found in S1 Data. AL, ad libitum; DR, dietary restriction; NOR, novel object recognition.
(TIF)

S2 Fig Dietary protein and Trp limit DR-induced memory performance. The body weight change (A, D), representative moving path (upper insets) and travel distance during the open field test (B, E), and times spent on objects during the NOR test (C, F) of young mice (2 months old) under dietary manipulations (AL, DR, and DR plus carb, protein, fat, Tyr, Trp, Cys, or Glu to a level equivalent to AL) for 8 weeks. Data are presented as mean ± SEM (*n* = 10–11 mice for each group). **P < 0.01; ***P < 0.001 by one-way ANOVA with Fisher’s LSD post hoc test. Underlying data can be found in S1 Data. AL, ad libitum; carb, carbohydrate; Cys, cysteine; DR, dietary restriction; Glu, glutamate; LSD, least significant difference; NOR, novel object recognition; Trp, tryptophan; Tyr, tyrosine.
(TIF)

S3 Fig DR affects serotonin metabolism and HTR6 protein expression in mouse prefrontal cortex. (A) Serotonin and 5-HIAA levels, (B) 5-HIAA/5-HT ratios, and (C) representative western blots and quantitative protein expression levels of HTR6 in the prefrontal cortex of AL and DR mice. Data are presented as mean ± SEM (*n* = 5–6 mice per group). *P < 0.05 by Student *t* test. Underlying data can be found in S1 Data. 5-HIAA, 5-hydroxyindoleacetic acid; 5-HT, 5-hydroxytryptamine; AL, ad libitum; DR, dietary restriction; HTR6, 5-hydroxytryptamine receptor 6.
(TIF)

S4 Fig Expression levels of HTRs, glucose metabolism and ketogenesis in WT and HTR6 KO mice. (A) Hippocampal mRNA expression levels of known HTRs, (B) representative western blots of hippocampal HTR6, (C) glucose tolerance test, (D) insulin tolerance test, and (E) nonfasted and 6-hour-fasted blood beta-hydroxybutyrate concentrations were measured in 4-month-old WT, heterozygous (“Het”), and/or homozygous (“Hom”) HTR6 KO mice. Data are presented as mean ± SEM (*n* = 6–9 mice for each group in A, C–E). ***P < 0.001 by Student *t* test or two-way ANOVA with Fisher’s LSD post hoc test. Underlying data can be found in S1 Data. HTR, 5-hydroxytryptamine receptor; KO, knockout; LSD, least significant difference; WT, wild type.
(TIF)

S5 Fig HTR6 is required for DR-induced structural alteration in DG hippocampal neurons. (A) Allen Brain Atlas of mRNA in situ (www.brainatlas.org) and (B) GENSAT of BAC–eGFP transgenic mice (www.gensat.org) show the HTR6 expression pattern. (C–I) Structural analyses of DG granule cells from WT and HTR6 KO mice fed on AL (blue) or DR (red) diet for 8 weeks. (C) Reconstructed DG granule cells. (D, E) The dendritic profiles (D) and dendritic length (E) of DG granule cells. (F, G) Reconstructed distal dendrites (F) and quantitative spine density (G) in distal (>150 μm from the soma) and proximal (<50 μm from the soma) dendrites of DG granule cells. (H, I) Representative micrographs (H) and quantitative cell density (I) from mouse hippocampal DG granule cells following Nissl staining. Data are presented as mean ± SEM (*n* = 40 cells, 26 dendritic segments, and 65–100 DG regions from 5–8 animals for each group). *P < 0.05, ***P < 0.001 by Student *t* test. (J) The input–output curves of brain slices from WT and HTR6 KO mice fed an AL (blue) or DR (red) diet for 8 weeks. Data are presented as mean ± SEM (*n* = 12–14 recordings per group). Underlying data can be found in S1 Data. AL, ad libitum; BAC–eGFP, bacterial artificial chromosome–enhanced green fluorescent protein; DG, dentate gyrus; DR, dietary restriction; GENSAT, Gene Expression Nervous System Atlas; HTR6, 5-hydroxytryptamine receptor 6; KO, knockout; WT, wild type.
(TIF)

S6 Fig mTOR activator and mTOR inhibitor treatments do not affect food intake, body weight, or locomotor activity of mice. (A) The daily food intake, (B) body weight change, and (C) travel distance during the open field test of AL, DR, and HTR6 KO mice on diets supplemented with mTOR activator (PA) or mTOR inhibitor (RA) for 8 weeks (*n* = 9–12 mice for each group). Data are presented as mean ± SEM. Underlying data can be found in S1 Data. AL, ad libitum; DR, dietary restriction; HTR6, 5-hydroxytryptamine receptor 6; KO, knockout; mTOR, mechanistic target of rapamycin; PA, phosphatidic acid; RA, rapamycin analog.
(TIF)

S1 Table Oligonucleotide primer sequences. (DOCX)

S2 Table Calculated food composition and food intake for experimental groups of mice. (DOCX)

S1 Data Underlying data for main figures and supporting figures. (XLSX)

## Abbreviations

5-HIAA: 5-hydroxyindoleacetic acid
5-HT: 5-hydroxytryptamine
AL: ad libitum
BAC: bacterial artificial chromosome–enhanced green fluorescent protein
Cdk5: cyclin-dependent kinase 5
CREB-1: cAMP-responsive element-binding protein 1
DG: dentate gyrus
DR: dietary restriction
GENSAT: Gene Expression Nervous System Atlas
GFP: green fluorescent protein
GR: glucocorticoid receptor
HTR6: 5-hydroxytryptamine receptor 6
IMS: intermittent metabolic switching
IP: intraperitoneal
KO: knockout
LTP: long-term potentiation
MR: mineralocorticoid receptor
mTOR: mechanistic target of rapamycin
mTORC1: mTOR complex 1
mTORC2: mTOR complex 2
NOR: novel object recognition
PA: phosphatidic acid
p-Akt: phosphorylated Akt
p-CREB: phosphorylated CREB
PKA: protein kinase A
p-PKA: phosphorylated PKA
p-S6K: phosphorylated S6K
RA: rapamycin analog
S6K: S6 kinase
SB: SB 399885 hydrochloride
WAY: WAY 208466 dihydrochloride
WT: wild type
