# Supplementary figures and images for: Serotonin receptor HTR6-mediated mTORC1 signaling regulates dietary restriction–induced memory enhancement
Source: PLoS Biol. 2019 Mar 18;17(3):e2007097. doi: 10.1371/journal.pbio.2007097 (PMC6438579; doi:10.1371/journal.pbio.2007097)

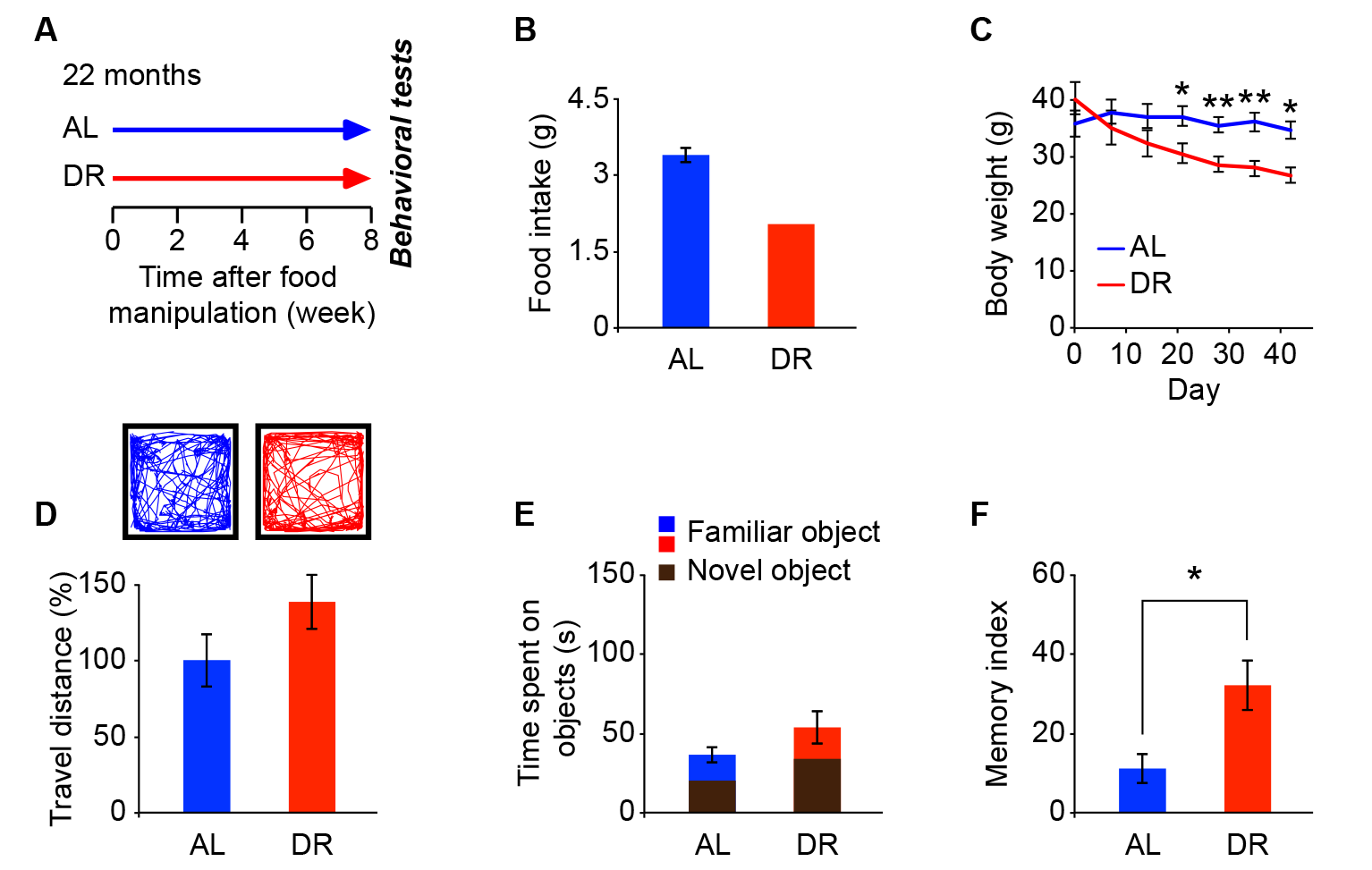

Supplement: S1 Fig — (A) The experimental diagram, (B) daily food intake, (C) body weight, (D) representative moving path (upper insets) and travel distance during the open field test, and (E) times spent on objects and (F) calculated memory indexes during the NOR test of aged mice (22 months old) under dietary manipulations (AL, blue; 60% food intake of AL [DR, red]) for 8 weeks (n = 9 mice per group). Data are presented as mean ± SEM. *P < 0.05; **P < 0.01 by Student t test. Underlying data can be found in S1 Data. AL, ad libitum; DR, dietary restriction; NOR, novel object recognition. (TIF) [file pbio.2007097.s001.tif]

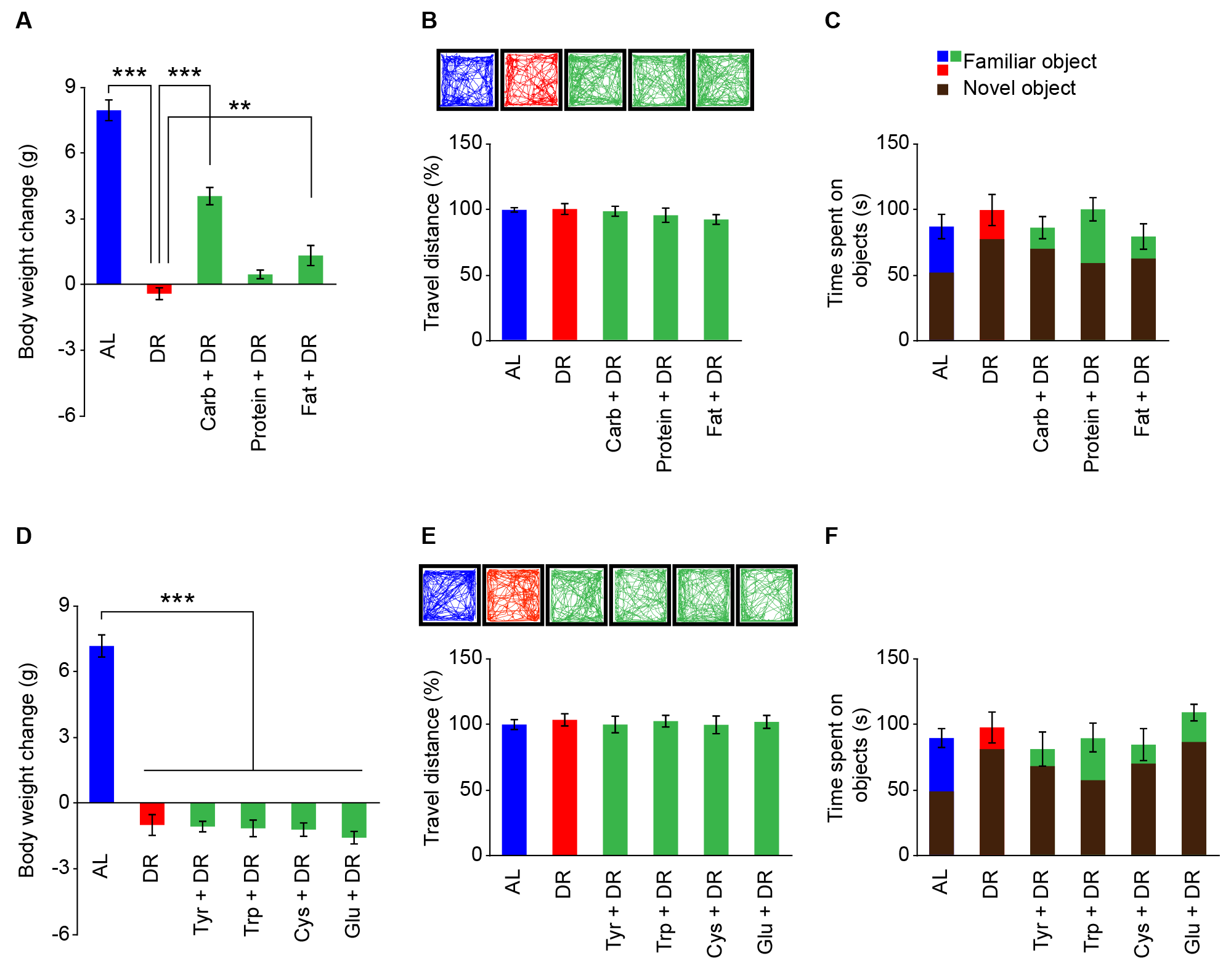

Supplement: S2 Fig — The body weight change (A, D), representative moving path (upper insets) and travel distance during the open field test (B, E), and times spent on objects during the NOR test (C, F) of young mice (2 months old) under dietary manipulations (AL, DR, and DR plus carb, protein, fat, Tyr, Trp, Cys, or Glu to a level equivalent to AL) for 8 weeks. Data are presented as mean ± SEM (n = 10–11 mice for each group). **P < 0.01; ***P < 0.001 by one-way ANOVA with Fisher’s LSD post hoc test. Underlying data can be found in S1 Data. AL, ad libitum; carb, carbohydrate; Cys, cysteine; DR, dietary restriction; Glu, glutamate; LSD, least significant difference; NOR, novel object recognition; Trp, tryptophan; Tyr, tyrosine. (TIF) [file pbio.2007097.s002.tif]

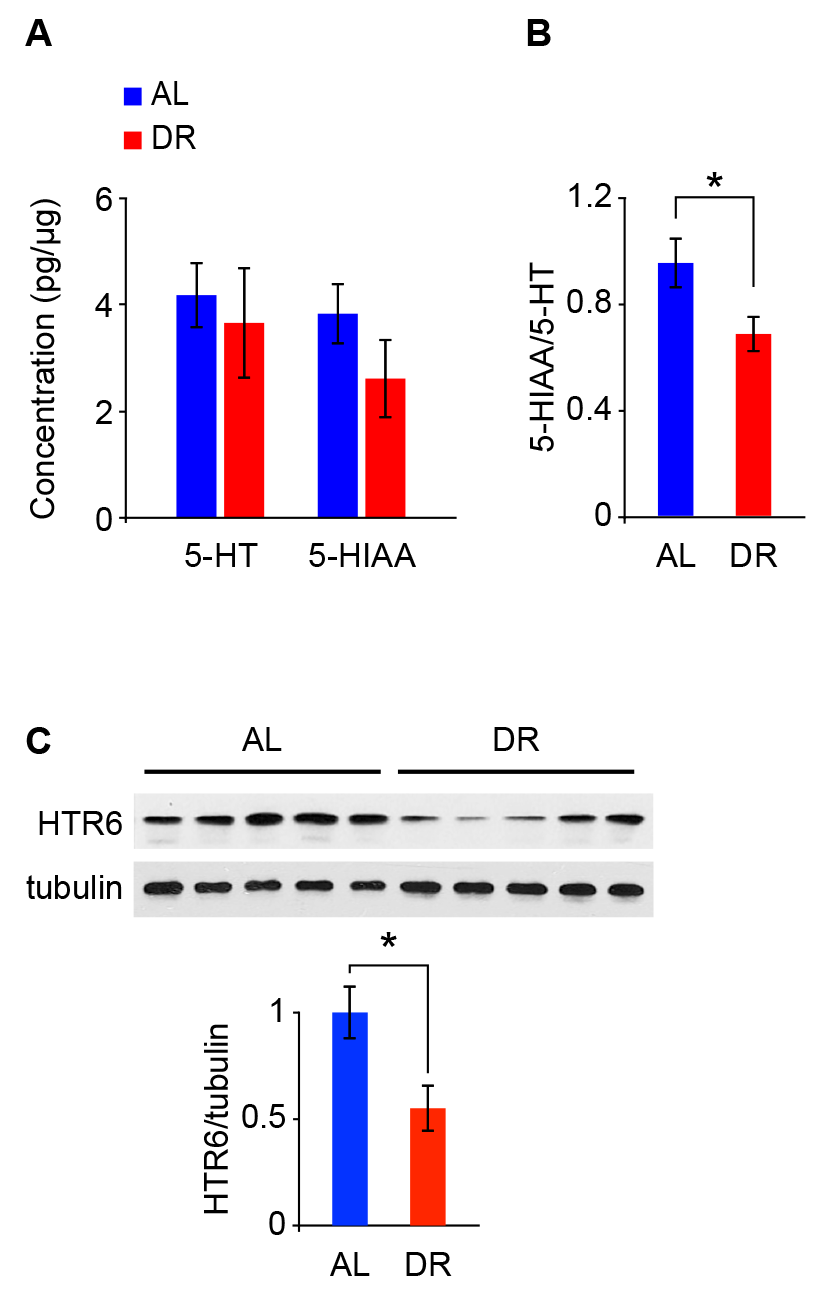

Supplement: S3 Fig — (A) Serotonin and 5-HIAA levels, (B) 5-HIAA/5-HT ratios, and (C) representative western blots and quantitative protein expression levels of HTR6 in the prefrontal cortex of AL and DR mice. Data are presented as mean ± SEM (n = 5–6 mice per group). *P < 0.05 by Student t test. Underlying data can be found in S1 Data. 5-HIAA, 5-hydroxyindoleacetic acid; 5-HT, 5-hydroxytryptamine; AL, ad libitum; DR, dietary restriction; HTR6, 5-hydroxytryptamine receptor 6. (TIF) [file pbio.2007097.s003.tif]

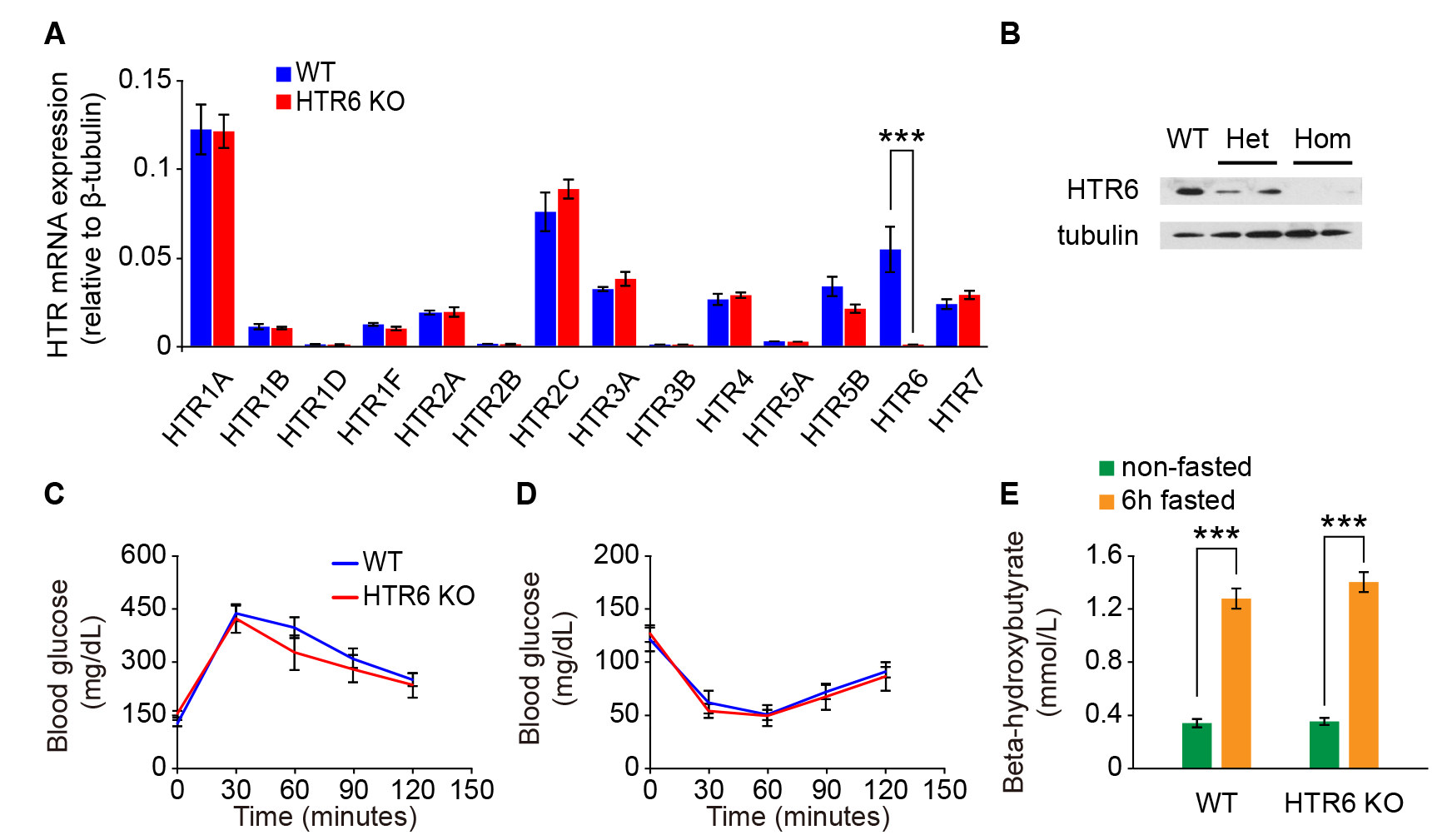

Supplement: S4 Fig — (A) Hippocampal mRNA expression levels of known HTRs, (B) representative western blots of hippocampal HTR6, (C) glucose tolerance test, (D) insulin tolerance test, and (E) nonfasted and 6-hour-fasted blood beta-hydroxybutyrate concentrations were measured in 4-month-old WT, heterozygous (“Het”), and/or homozygous (“Hom”) HTR6 KO mice. Data are presented as mean ± SEM (n = 6–9 mice for each group in A, C–E). ***P < 0.001 by Student t test or two-way ANOVA with Fisher’s LSD post hoc test. Underlying data can be found in S1 Data. HTR, 5-hydroxytryptamine receptor; KO, knockout; LSD, least significant difference; WT, wild type. (TIF) [file pbio.2007097.s004.tif]

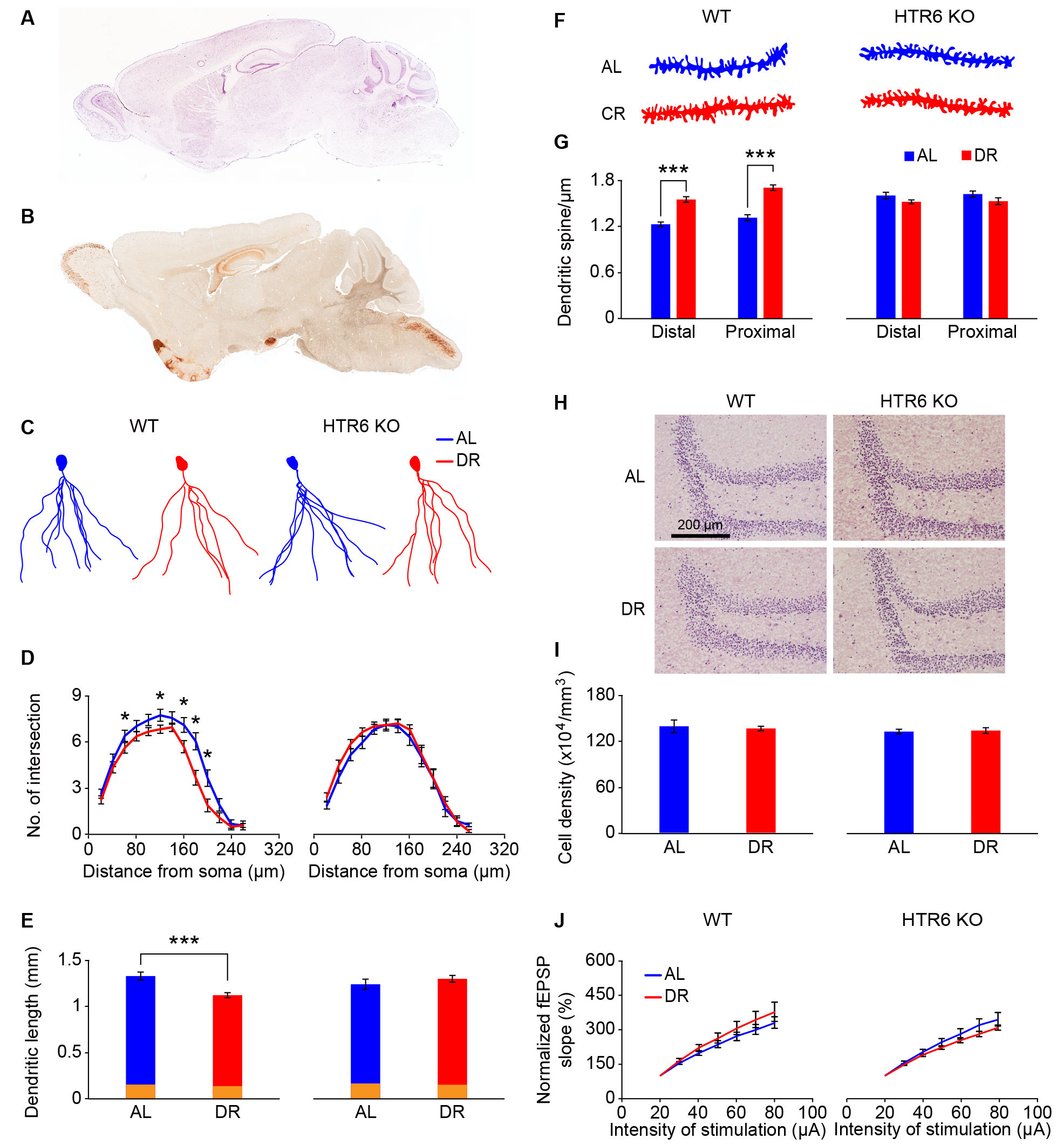

Supplement: S5 Fig — (A) Allen Brain Atlas of mRNA in situ (www.brainatlas.org) and (B) GENSAT of BAC–eGFP transgenic mice (www.gensat.org) show the HTR6 expression pattern. (C–I) Structural analyses of DG granule cells from WT and HTR6 KO mice fed on AL (blue) or DR (red) diet for 8 weeks. (C) Reconstructed DG granule cells. (D, E) The dendritic profiles (D) and dendritic length (E) of DG granule cells. (F, G) Reconstructed distal dendrites (F) and quantitative spine density (G) in distal (>150 μm from the soma) and proximal (<50 μm from the soma) dendrites of DG granule cells. (H, I) Representative micrographs (H) and quantitative cell density (I) from mouse hippocampal DG granule cells following Nissl staining. Data are presented as mean ± SEM (n = 40 cells, 26 dendritic segments, and 65–100 DG regions from 5–8 animals for each group). *P < 0.05, ***P < 0.001 by Student t test. (J) The input–output curves of brain slices from WT and HTR6 KO mice fed an AL (blue) or DR (red) diet for 8 weeks. Data are presented as mean ± SEM (n = 12–14 recordings per group). Underlying data can be found in S1 Data. AL, ad libitum; BAC–eGFP, bacterial artificial chromosome–enhanced green fluorescent protein; DG, dentate gyrus; DR, dietary restriction; GENSAT, Gene Expression Nervous System Atlas; HTR6, 5-hydroxytryptamine receptor 6; KO, knockout; WT, wild type. (TIF) [file pbio.2007097.s005.tif]

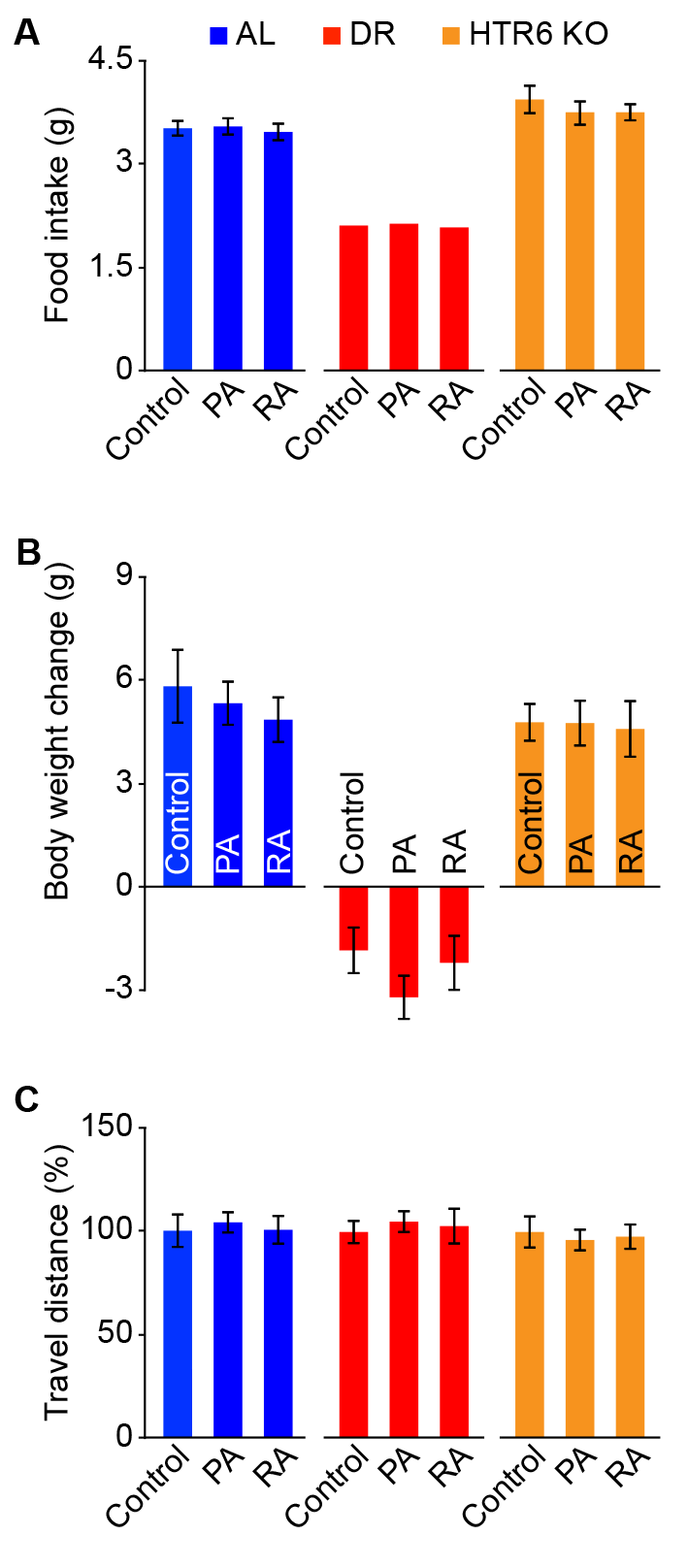

Supplement: S6 Fig — (A) The daily food intake, (B) body weight change, and (C) travel distance during the open field test of AL, DR, and HTR6 KO mice on diets supplemented with mTOR activator (PA) or mTOR inhibitor (RA) for 8 weeks (n = 9–12 mice for each group). Data are presented as mean ± SEM. Underlying data can be found in S1 Data. AL, ad libitum; DR, dietary restriction; HTR6, 5-hydroxytryptamine receptor 6; KO, knockout; mTOR, mechanistic target of rapamycin; PA, phosphatidic acid; RA, rapamycin analog. (TIF) [file pbio.2007097.s006.tif]
