# Supplementary material for: Serotonin receptor HTR6-mediated mTORC1 signaling regulates dietary restriction–induced memory enhancement
Source: PLoS Biol. 2019 Mar 18;17(3):e2007097. doi: 10.1371/journal.pbio.2007097 (PMC6438579; doi:10.1371/journal.pbio.2007097)
Supplement: S1 Table — (DOCX) [file pbio.2007097.s007.docx]

**S1 Table.** Oligonucleotide primer sequences.

| Primer | Sequence | Product size(bp) |
| --- | --- | --- |
| HTR1A-F  HTR1A-R | 5’-GGGCATTGCTCTTGTTACTTTGA-3’  5’-GTGCGGCATGTTGCACTTAGT-3’ | 120 |
| HTR1B-F  HTR1B-R | 5’-CTGGTTTCACATGGCCATTTTT-3’  5’-ATCAGTTTGTGGAACGCTTGTTT-3’ | 120 |
| HTR1D-F  HTR1D-R | 5’-GCCTTCTGGTCTACCTGGGATT-3’  5’-AATCAATGAGCTCCAGGAGATGA-3’ | 120 |
| HTR1F-F  HTR1F-R | 5’-AGATATGAAGCTCAGCGGCTTT-3’  5’-AAGATTGCAAATGTGCAGAAAATG-3’ | 120 |
| HTR2A-F  HTR2A-R | 5’-TGCCCATTCTTCATCACCAATAT-3’  5’-GTTGACGGCTGAGGAGAGATAAC-3’ | 120 |
| HTR2B-F  HTR2B-R | 5’-CGAGCCACAAAGTCAGTAAAAGC-3’  5’-AGGGTTGATCCCATTTCGAATT-3’ | 120 |
| HTR2C-F  HTR2C-R | 5’-CACACCTTGGAGTGAGCCTTTTA-3’  5’-TCCTTGGCACACTGTTGAATTTA-3’ | 120 |
| HTR3A-F  HTR3A-R | 5’-GGTCACTCTCTGGTCCATTTGG-3’  5’-GGAAACAGGATGCAGGACCTAAG-3’ | 120 |
| HTR3B-F  HTR3B-R | 5’-GCAGCAACTGCGTACACTTAGGT-3’  5’-TTGGAAGGTAGAGGCTGGAATGT-3’ | 120 |
| HTR4-F  HTR4-R | 5’-ATGCTGAGATGGTTCGTGTCAA-3’  5’-TGATCCCGCTACAACGTCAGTAT-3’ | 120 |
| HTR5A-F  HTR5A-R | 5’-AGGAGCTACAGCAGTGCTTTCAA-3’  5’-TGCAAAGGTTCATGGGACAATAT-3’ | 120 |
| HTR5B-F  HTR5B-R | 5’-GGATCTTGATTGGCGTGTTTGT-3’  5’-ATCCAAGCCACAGGAATATGCTT-3’ | 120 |
| HTR6-F  HTR6-R | 5’-CATCCTGCTGAGCATGTTCTTT-3’  5’-CTATTACAGTATCCCAGCCATGTGA-3’ | 126 |
| HTR7-F  HTR7-R | 5’-CACTTGAACCCCCATTGTTAATACT-3’  5’-GGAGTTAAGGGACAGAATTCATCAG-3’ | 120 |
| β tubulin-F  β tubulin-R | 5’-GTCCTTTTGGCCAGATCTTCAG-3’  5’-CATCCAAGACAGAGTCAACCAACT-3’ | 120 |
| HTR6 (WT)-F  HTR6 (WT)-R | 5’-CCATTCTCAACCTCTGCCTCAT-3’  5’-GCAAGATCCTGCAGTAGGTGAA-3’ | 300 |
| HTR6 (KO)-F  HTR6 (KO)-R | 5’-TGGGTAATAAGCGTTGGCAATTT-3’  5’-AGTTTTCTTGCGGCCCTAATCC-3’ | 465 |

The forward and reverse primers used for RT-PCR detection of the listed genes are indicated by “-F” and “-R”, respectively.
