# Supplementary material for: Serotonin receptor HTR6-mediated mTORC1 signaling regulates dietary restriction–induced memory enhancement
Source: PLoS Biol. 2019 Mar 18;17(3):e2007097. doi: 10.1371/journal.pbio.2007097 (PMC6438579; doi:10.1371/journal.pbio.2007097)
Supplement: S2 Table — (DOCX) [file pbio.2007097.s008.docx]

| Ingredient (g) | AL | DR | DR+Carb | DR+Protein | DR+Fat | DR+Trp | | DR+Tyr | DR+Glu | DR+Cys |
| --- | --- | --- | --- | --- | --- | --- | --- | --- | --- | --- |
| Corn starch | 1.373 | 0.824 | 1.373 | 0.824 | 0.824 | 0.824 | | 0.824 | 0.824 | 0.824 |
| Sucrose | 0.345 | 0.207 | 0.345 | 0.207 | 0.207 | 0.207 | | 0.207 | 0.207 | 0.207 |
| Dextrin | 0.456 | 0.274 | 0.456 | 0.274 | 0.274 | 0.274 | | 0.274 | 0.274 | 0.274 |
| Casein | 0.691 | 0.414 | 0.414 | 0.691 | 0.414 | 0.414 | | 0.414 | 0.414 | 0.414 |
| L-Cystine | 0.010 | 0.006 | 0.006 | 0.010 | 0.006 | 0.006 | | 0.006 | 0.006 | 0.006 |
| Soybean oil | 0.242 | 0.145 | 0.145 | 0.145 | 0.242 | 0.145 | | 0.145 | 0.145 | 0.145 |
| Fiber (cellulose) | 0.173 | 0.104 | 0.104 | 0.104 | 0.104 | 0.104 | | 0.104 | 0.104 | 0.104 |
| Mineral | 0.121 | 0.073 | 0.073 | 0.073 | 0.073 | 0.073 | | 0.073 | 0.073 | 0.073 |
| Vitamin | 0.035 | 0.021 | 0.021 | 0.021 | 0.021 | 0.021 | | 0.021 | 0.021 | 0.021 |
| TBHQ | 4.84E-05 | 2.90E-05 | 2.90E-05 | 2.90E-05 | 2.90E-05 | 2.90E-05 | | 2.90E-05 | 2.90E-05 | 2.90E-05 |
| Choline Bitartrate | 0.009 | 0.005 | 0.005 | 0.005 | 0.005 | 0.005 | | 0.005 | 0.005 | 0.005 |
| Tryptophan |  |  |  |  |  | 0.003 | |  |  |  |
| Tyrosine |  |  |  |  |  |  | 0.014 | |  |  |
| Glutamate |  |  |  |  |  |  |  | | 0.056 |  |
| L-Cystine |  |  |  |  |  |  |  | |  | 0.004 |
| Food intake (g/day) | 3.454 | 2.072 | 2.942 | 2.353 | 2.169 | 2.075 | 2.086 | | 2.129 | 2.077 |

**S2 Table. Calculated food composition and food intake for experimental groups of mice.**

Food composition is based on the AIN-93G purified diet which contains 39.75% corn starch, 10% sucrose, 13.2% maltodextrin, 20% casein, 7% soybean oil, 5% fiber (cellulose), 3.5% mineral mix (AIN-93G-MX), 1% vitamin mix (AIN-93G-VX), 0.25% choline bitartrate and 0.0014% tertiary butylhydroquinone (TBHQ). The amino acid composition of the AIN-93G purified diet is calculated to be 0.21% tryptophan (trp), 0.98% tyrosine (tyr), 4.08% glutamate (glu) and 0.30% cysteine (cys).
